# Long-term ketamine infusion-induced cholestatic liver injury in COVID-19-associated acute respiratory distress syndrome

**DOI:** 10.1186/s13054-022-04019-8

**Published:** 2022-05-23

**Authors:** Pedro David Wendel-Garcia, Rolf Erlebach, Daniel Andrea Hofmaenner, Giovanni Camen, Reto Andreas Schuepbach, Christoph Jüngst, Beat Müllhaupt, Jan Bartussek, Philipp Karl Buehler, Rea Andermatt, Sascha David

**Affiliations:** 1 Institute of Intensive Care Medicine, University Hospital Zurich, Rämistrasse 100, 8091 Zurich, Switzerland; 2 Department of Gastroenterology and Hepatology, University Hospital Zurich, Zurich, Switzerland; 3 Department of Quantitative Biomedicine, University of Zurich, Zurich, Switzerland

**Keywords:** Hypnotics and sedatives, Chemical and drug-induced liver injury, Cholestasis, Cholangitis, Cholangiopathy

## Abstract

**Background:**

A higher-than-usual resistance to standard sedation regimens in COVID-19 patients suffering from acute respiratory distress syndrome (ARDS) has led to the frequent use of the second-line anaesthetic agent ketamine. Simultaneously, an increased incidence of cholangiopathies in mechanically ventilated patients receiving prolonged infusion of high-dose ketamine has been noted. Therefore, the objective of this study was to investigate a potential dose–response relationship between ketamine and bilirubin levels.

**Methods:**

Post hoc analysis of a prospective observational cohort of patients suffering from COVID-19-associated ARDS between March 2020 and August 2021. A time-varying, multivariable adjusted, cumulative weighted exposure mixed-effects model was employed to analyse the exposure–effect relationship between ketamine infusion and total bilirubin levels.

**Results:**

Two-hundred forty-three critically ill patients were included into the analysis. Ketamine was infused to 170 (70%) patients at a rate of 1.4 [0.9–2.0] mg/kg/h for 9 [4–18] days. The mixed-effects model revealed a positively correlated infusion duration–effect as well as dose–effect relationship between ketamine infusion and rising bilirubin levels (*p* < 0.0001). In comparison, long-term infusion of propofol and sufentanil, even at high doses, was not associated with increasing bilirubin levels (*p* = 0.421, *p* = 0.258). Patients having received ketamine infusion had a multivariable adjusted competing risk hazard of developing a cholestatic liver injury during their ICU stay of 3.2 [95% confidence interval, 1.3–7.8] (*p* = 0.01).

**Conclusions:**

A causally plausible, dose–effect relationship between long-term infusion of ketamine and rising total bilirubin levels, as well as an augmented, ketamine-associated, hazard of cholestatic liver injury in critically ill COVID-19 patients could be shown. High-dose ketamine should be refrained from whenever possible for the long-term analgosedation of mechanically ventilated COVID-19 patients.

**Supplementary Information:**

The online version contains supplementary material available at 10.1186/s13054-022-04019-8.

## Background

The coronavirus disease 2019 (COVID-19) pandemic has incepted a surge of mechanically ventilated patients suffering from acute respiratory distress syndrome (ARDS), who have demonstrated to be characteristically complex to sedate and require high sedative and analgesic doses [1].

In order to ensure deep sedation, a requirement for lung protective mechanical ventilation, standard first-line sedative and analgesic agents, such as propofol, midazolam and opioids, have frequently required combination with former second-line drugs, which are only seldom employed for long-term sedation in other scenarios [1, 2]. Ketamine, an *N-methyl-D-aspartate* (NMDA) receptor antagonist, due to its combined sedative and analgesic properties, has rapidly transitioned to a routinely employed anaesthetic agent in many intensive care units treating patients suffering from COVID-19 (Additional file 1: e-Table 1).

Evidence on the systemic effects of long-term infusion of ketamine is limited; however, its prolonged infusion in doses above 1 mg/kg/h has been associated with hepatotoxicity [3], leading the French National Agency for the Safety of Medicine to issue an alert cautioning against its use in high doses [4]. Recently, an increasing incidence of cholangiopathies, including secondary sclerosing cholangitis (SSC), has been noted in mechanically ventilated COVID-19 patients receiving long-term infusion of ketamine [5–8].

Considering the growing number of reports, combined with the clinical impression of a progressive cholestatic liver injury in mechanically ventilated COVID-19 patients receiving ketamine, its role as an inductor and/or exacerbator of cholestatic liver injury has gained plausibility. Therefore, the main objective of this study was to investigate a causal relationship between the long-term, high-dose infusion of ketamine and the occurrence of cholestatic liver injury in mechanically ventilated patients suffering from COVID-19-associated ARDS.

## Methods

This study was planned as a post hoc analysis of a prospective observational study conducted at the Institute of Intensive Care Medicine of the University Hospital Zurich, an academic tertiary referral centre, between March 2020 and August 2021. Both the prospective study protocol (ClinicalTrials.gov Identifier: NCT04410263, BASEC ID: ZH 2020-00646) and the post hoc analysis protocol (BASEC ID: ZH 2021-01617) were approved by the Swiss regional cantonal ethical commission. The study complies with the Declaration of Helsinki, the Guidelines on Good Clinical Practice (GCP-Directive) issued by the European Medicines Agency, the Swiss law and Swiss regulatory authority requirements and has been designed in accordance with the Strengthening the Reporting of Observational Studies in Epidemiology (STROBE) guidelines for observational studies.

### Population

All consecutively admitted, invasively mechanically ventilated patients, above 18 years of age, with a confirmed SARS-CoV-2 infection by real-time reverse transcriptase polymerase chain reaction, suffering from COVID-19-associated ARDS according to the Berlin definition were included [9]. Patients having solely been treated by means of non-invasive respiratory support or having denied informed consent for this study were excluded. The period of observation began either with the endotracheal intubation and concomitant analgosedation of the patient or with the admission of the already intubated and analgosedated patient. The observation period ended with the patients’ discharge from the intensive care unit (ICU).

### Sedation and ventilation strategy

Patients were treated according to an institute-specific guideline tailored for COVID-19 management. Primarily, sedation was approached through the combined use of propofol and sufentanil. Secondarily, in order to minimize patient–ventilator dys-synchrony and to ensure lung protective ventilation, clonidine, ketamine, midazolam and/or volatile anaesthetics could be considered according to the attending physician’s choice.

Patients were mechanically ventilated targeting a tidal volume below 6 ml/kg ideal body weight, a plateau pressure ≤ 30 cmH_2_O and a driving pressure ≤ 15 cmH_2_O, under permissive hypercapnia with a lower pH limit of 7.25, in order to ensure an arterial haemoglobin saturation above 88%. Additionally, institutional guidelines indicated prone position for patients with a partial pressure of arterial oxygen (paO_2_) over inspired oxygen fraction (FiO_2_) ratio (P/F ratio) < 200 mmHg and the use of neuromuscular blocking agents in patients with a P/F ratio < 150 mmHg or presenting an uncontrollable respiratory drive and vigorous breathing efforts under deep sedation.

### Data collection

The clinical and laboratory parameter collection was planned and prescribed prospectively, leading to a minimal amount of missing data (Additional file 1: e-Table 2); consequently, we assumed a missing at random mechanism for missing values. Measurements of inflammatory parameters, including C-reactive protein and procalcitonin, as well as haematological blood counts, arterial blood gas analyses and markers of hepatic as well as renal organ dysfunction, most prominently bilirubin and creatinine, were performed daily between 6:00 and 8:00 a.m. Clinical data comprising vitals, ventilation parameters and drug infusions were continuously recorded by an automated patient data management system, and representative values were extracted daily between 6:00 and 8:00 a.m. Drug doses, including vasopressor doses, were exclusively modelled in the form of daily-cumulated doses in order to enable a robust modelling of the effective daily requirements.

### Definition of cholestatic liver injury

In accordance with the European Association for the Study of the Liver (EASL) clinical practice guidelines and the Council for International Organizations of Medical Sciences (CIOMS) criteria of drug-induced liver disorders, an acute drug-induced cholestatic liver injury was defined as a alkaline phosphatase > 1.5 times the upper limit of normality (ULN) and a gamma-glutamyltransferase > 3 times the ULN [10, 11]. Patients, who additionally presented a liver dysfunction, defined as a bilirubin > 2 times the ULN, were considered to present a severe cholestatic liver injury [10, 12]. The ratio of alanine aminotransferase to alkaline phosphatase (both normalized to their respective ULN’s) can be further used to discriminate between a predominantly hepatocellular (> 5) and a chiefly cholestatic liver injury (< 2) [10]. Bilirubin was employed as primary, longitudinal surrogate for cholestatic liver injury, as it defines liver dysfunction in drug-induced liver injury and in the Sequential Organ Failure Assessment (SOFA) score [10, 13].

### Causal model and cumulative drug exposure model

In order to ensure a well-defined causal model to study the potential effect of ketamine infusion on cholestatic liver injury, surrogately represented by the total bilirubin levels in blood, a structural causal model was graphically specified employing directed acyclic graphs based on previous literature [5–8, 14]. In a first step, a time-independent causal model was drafted including all relevant variables possibly affecting the investigated causal relationship; see Additional file 1: e-Appendix 1. Subsequently, the model was expanded to fit the time-varying properties underlying the modelled causal pathway; see Additional file 1: e-Appendix 2.

The impact of a drug infused over a long period can usually not be described by the isolated dose applied at a certain time point, as the effect is generally likely to cumulate over time [15]. In order to model the complex time-dependent causal relationships between a drug exposure and an effect, the *weighted cumulative exposure* (WCE) approach was recently proposed [16, 17]. This model combined with a linear mixed-effects model corrects the time-varying exposure–effect relationship for the daily varying status of the patients, compensating for differences in evolution of patients, thus enabling statistically unbiased inference of the investigated exposure–effect interaction. For further details on the WCE approach and its implementation in the framework of this study, refer to Additional file 1: e-Appendix 3.

### Statistical analysis

Comparisons of population characteristics were made using the Wilcoxon signed-rank and the chi-squared test, as appropriate. The association between total cumulative exposure to ketamine and maximal bilirubin levels during the ICU stay was modelled by means of univariable and multivariable linear regression. Bilirubin was modelled as an unpenalized cubic B-spline regression. For the multivariable adjusted model, covariates were chosen based on the previously described directed acyclic graphs, and the worst values recorded during the ICU stay were employed.

Flexible modelling of the time-varying effect of ketamine on repeated-measures of total bilirubin levels was approached by means of a linear mixed-effects WCE model [17]. Daily bilirubin measurements were entered as outcome variable into the model; daily ketamine, propofol and sufentanil doses were entered as independent fixed effects, both modelled as WCEs; additionally all variables specified in the directed acyclic graphs were implemented as daily time-varying covariates; and finally per patient random intercepts and intra-patient random slopes were entered into the model. Interaction terms were tested and retained only if they were found to contribute to the model. Additionally, a first-order autoregressive term was tested and omitted, as it did not improve model fit. P values for individual fixed effects were obtained by Satterthwaite’s degrees of freedom method. As sensitivity analysis, the same linear mixed-effects WCE model was specified for alkaline phosphatase as an outcome variable and for a sub-cohort of patients not having received extracorporeal membrane oxygenation (ECMO).

Finally, the effect of ketamine infusion, as binary classifier, on the incidence of cholestatic and severe cholestatic liver injury, as above defined, was modelled by means of a fine and grey competing risk proportional hazards model considering death as a competing event [18]. Mortality, on the other hand, was modelled by means of a Cox proportional hazard model. Univariable and multivariable adjusted models were both generated, adjusting for the previously described covariates at the time point of endotracheal intubation. Proportional hazard assumptions were assessed through inspection of Schoenfeld residuals. Time-to-event plots were generated by implementing the Kaplan–Meier estimator.

Statistical analysis was performed through a fully scripted data management pathway using the R environment for statistical computing version 4.0.2 [19]. Due to the observational, prospective nature of this cohort study, no power calculations were performed [20]. A two-sided *p* < 0.05 was considered statistically significant. Values are given as medians with interquartile ranges (IQRs) or counts and percentages as appropriate.

## Results

### Overall

**Fig. 1:**
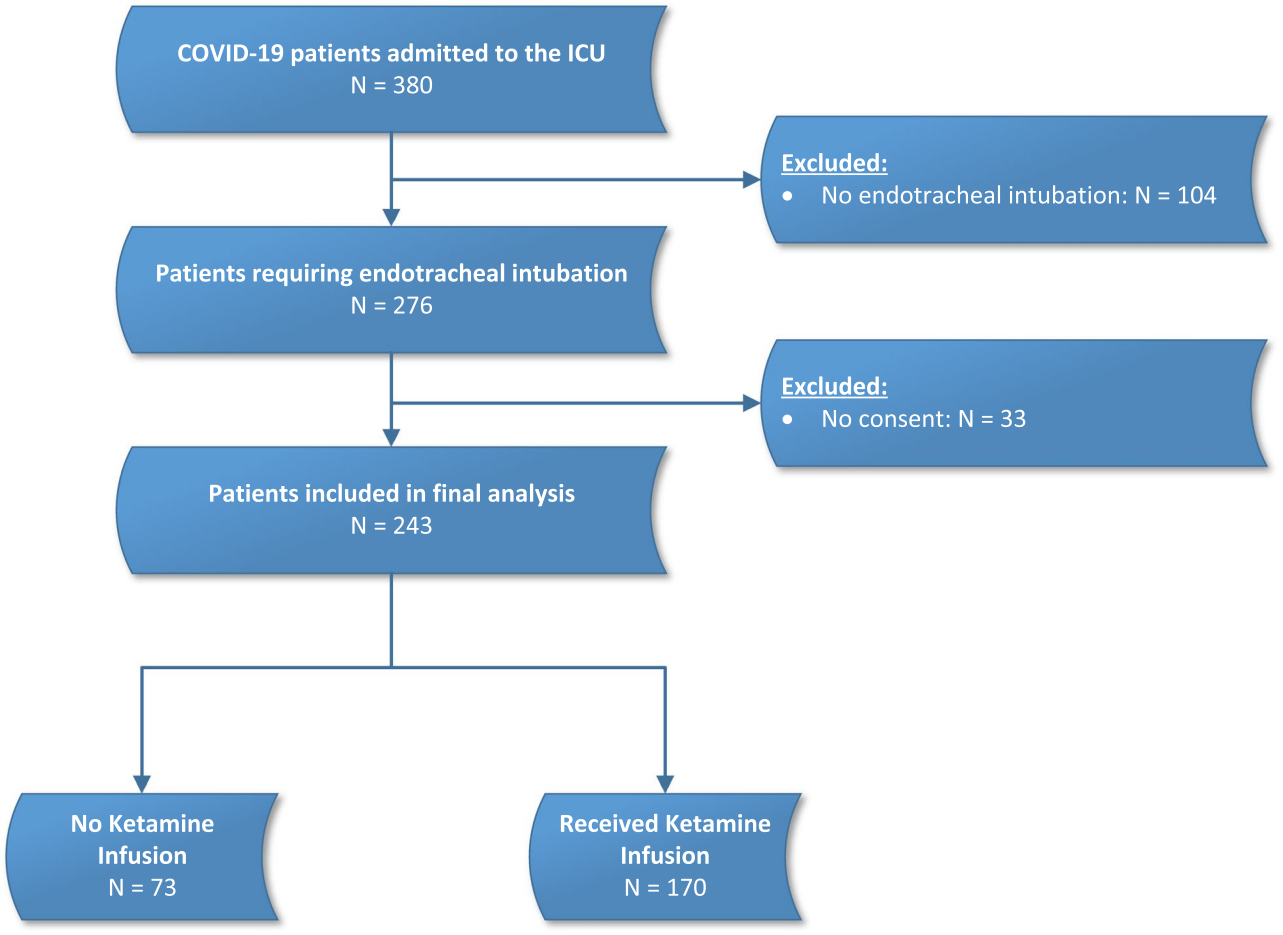
Study flow chart

From March 2020 until August 2021, 380 critically ill patients suffering COVID-19 were admitted to the Institute of Intensive Care Medicine of the University Hospital Zurich, of these 276 required endotracheal intubation and mechanical ventilation and 243 were included into the final analysis (Fig. 1).

Overall, patients were mainly male (67%), aged 64 [55–70] years and presented with a body mass index (BMI) of 28 [25–32] kg/m^2^ (Table 1). Patients were admitted to the hospital a median of 6 [2–9] days after symptom onset and had to be transferred to the ICU 2 [0–7] days later, where they presented with a Sequential Organ Failure Assessment (SOFA) score of 12 [9–14].

**Table 1:** Baseline characteristics at intensive care unit admission and at the time point of intubation

	Overall	No ketamine infusion	Ketamine infusion	*p* value
n	243	73	170	
**Characteristics at intensive care unit admission**				
Age, years	64 [55–70]	66 [57–72]	62 [54–68]	0.066
Sex, male	164 (67)	52 (71)	112 (66)	0.505
Body mass index, kg/m^2^	28 [25–32]	28 [24–31]	28 [25–33]	0.273
Time from symptoms until hospital admission, days	6 [2–9]	4 [0–10]	6 [2–9]	0.508
Time from hospital admission until ICU admission, days	2 [0–7]	2.0 [0–6]	2 [0–7]	0.556
SAPS II score at ICU admission	55 [44–64]	54 [43–66]	55 [44–63]	0.806
APACHE II score at ICU admission	24 [21–28]	24 [21–28]	24 [21–28]	0.483
SOFA score at ICU admission	12 [9–14]	12 [9–14]	13 [9–14]	0.240
Comorbidities				
Arterial hypertension	69 (28)	21 (29)	48 (28)	1.000
Diabetes mellitus	55 (23)	15 (21)	40 (24)	0.732
Ischemic heart disease	30 (12)	9 (12)	21 (12)	1.000
Chronic heart failure	13 (5)	5 (7)	8 (5)	0.712
Chronic kidney disease (moderate to severe)	33 (14)	12 (16)	21 (12)	0.517
Chronic liver disease (mild)	2 (1)	1 (1)	1 (1)	1.000
Chronic liver disease (moderate to severe)	8 (3)	4 (5)	4 (2)	0.390
Immunosuppression	19 (8)	7 (10)	12 (7)	0.680
**Characteristics at the time point of intubation**				
Time from ICU admission until intubation, days	0 [0–1]	0 [0–0]	0 [0–1]	0.950
SOFA score at intubation	13 [11–15]	12 [11–14]	14 [12–15]	0.003
PaO_2_/FiO_2_ Ratio, mmHg	146 [104–193]	168 [116–229]	140 [97–176]	< 0.001
Static respiratory system compliance, ml/cmH_2_O	34 [24–45]	35 [29–50]	33 [22–43]	0.088
Norepinephrine dose, μg/kg/min	0.1 [0.0–0.2]	0.1 [0.0–0.2]	0.1 [0.0–0.2]	0.460
Arterial lactate, mmol/l	1.4 [1.0–1.9]	1.4 [1.0–2.1]	1.3 [1.0–1.8]	0.391
Leucocyte count, 10^9^/l	11.2 [7.9–15.7]	10.9 [7.3–15.5]	11.4 [8.0–15.8]	0.536
Neutrophil count, 10^9^/l	9.2 [56.3–13.6]	8.9 [5.8–13.0]	9.4 [6.4–13.9]	0.416
Lymphocyte count, 10^9^/l	1.8 [1.2–2.6]	1.8 [1.2–2.7]	1.7 [1.2–2.6]	0.591
Thrombocyte count, 10^9^/l	238 [178–309]	240 [178–310]	235 [178–305]	0.932
C-reactive protein, mg/l	121 [59–213]	83.0 [42–187]	135 [66–234]	0.012
Procalcitonin, μg/l	0.5 [0.2–2.1]	0.7 [0.2–3.4]	0.5 [0.3–1.8]	0.159
Interleukin-6, ng/l	314 [170–861]	458 [251–1002]	268 [164–775]	0.066
D-dimer, mg/l	2530 [1150–5450]	2233 [1038–6508]	2540 [1180–4660]	0.947
Creatinine, μmol/l	85 [64–123]	83 [63–121]	85 [64–129]	0.900
Aspartate aminotransferase, U/l	49 [33–84]	45 [31–67]	51 [35–89]	0.117
Alanine aminotransferase, U/l	41 [26–71]	33 [21–67]	43 [29–72]	0.058
Gamma-glutamyltransferase, U/l	98 [49–234]	67 [35–114]	121 [56–273]	< 0.001
Alkaline phosphatase, U/l	80 [62–116]	73 [58–107]	85 [63–120]	0.155
Bilirubin (total), μmol/l	5 [3–7]	5 [4–7]	4 [3–6]	0.195
International normalized ratio	1.0 [1.0–1.1]	1.1 [1.0–1.1]	1.0 [1.0–1.1]	< 0.001
**Characteristics of ketamine infusion**				
Time from intubation until ketamine infusion, days	0 [0–2]	–	0 [0–2]	< 0.001
Duration of ketamine infusion, days	5 [0–13]	0 [0–0]	9 [4–18]	< 0.001
Dose of ketamine infusion, mg/kg/h	1.0 [0.0–1.7]	0 [0–0]	1.4 [0.9–2.0]	< 0.001

Quantitative data are expressed as median [interquartile range] or counts (percentages) as appropriate

*APACHE II* Acute Physiology and Chronic Health Disease Classification System, *FiO*_*2*_ fraction of inspired oxygen, *ICU* intensive care unit, *PaO*_*2*_ partial pressure of arterial oxygen, *SAPS II* Simplified Acute Physiology Score II, *SOFA* Sequential Organ Failure Assessment

### Intubation, mechanical ventilation and ketamine infusion

Patients required endotracheal intubation a median of 0 [0–1] days after ICU admission and were characterized by a P/F ratio of 146 [104–193] and a static respiratory system compliance of 34 [24–45] ml/cmH_2_O (Table 1).

One-hundred seventy (70%) patients received ketamine as a second-line sedation agent, a median of 0 [0–2] days after intubation. Ketamine was infused at a rate of 1.4 [0.9–2.0] mg/kg/h over 9 [4–18] days. Patients who received ketamine presented with a higher SOFA score at intubation (ketamine: 14 [12–15] vs. no ketamine: 12 [11–14], *p* = 0.003), but not at ICU admission (*p* = 0.240), as well as a lower P/F ratio (140 [97–176] mmHg) than patients who did not receive ketamine (168 [116–229] mmHg, *p* < 0.001).

### Cumulative ketamine dose and maximal bilirubin

**Fig. 2:**
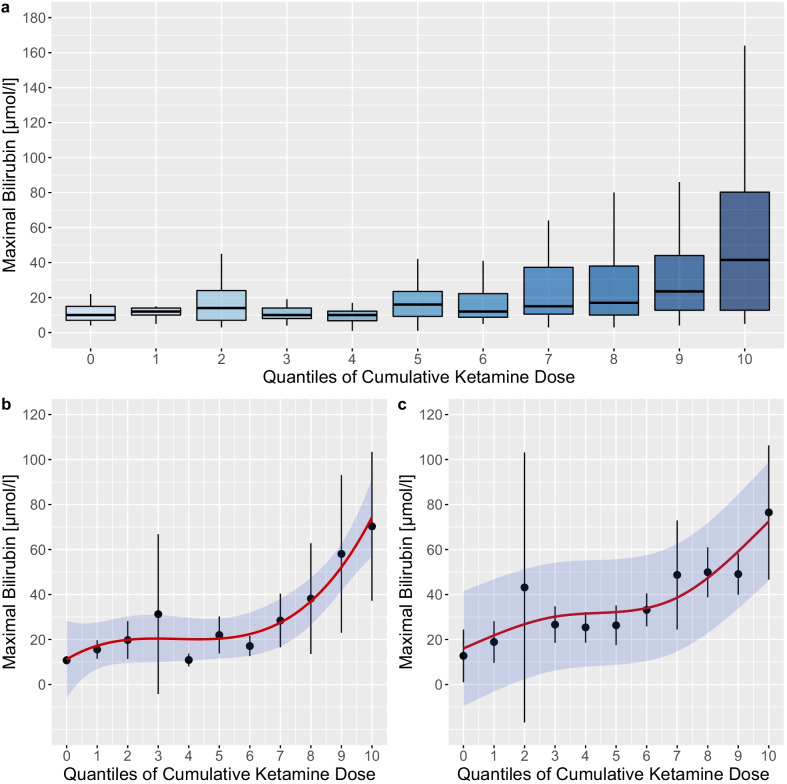
Association between quantiles of cumulatively infused ketamine and maximal bilirubin levels during mechanical ventilation. **a** Box plots presenting the distribution of maximal bilirubin levels during mechanical ventilation at different quantiles of cumulatively infused ketamine. **b** Unadjusted B-spline regression between maximal bilirubin levels and quantiles of cumulative ketamine. **c** Multivariable adjusted B-spline regression between maximal bilirubin levels and quantiles of cumulative ketamine. Worst values during the mechanical ventilation period were employed for all covariates in the model. Red lines represent regression estimates and the blue-shaded area 95% confidence intervals

Sub-dividing the cohort of 243 patients into 11 quantiles of cumulative ketamine dose over their period of intubation visually (Fig. 2a) and in a splined logistic regression (Fig. 2b, Additional file 1: e-Table 3) revealed an association between higher doses of ketamine and a higher maximal bilirubin level during the ICU stay (*p* < 0.0001). This effect remained for the quantiles 7 to 10 after multivariable adjustment (*p* < 0.001) (Fig. 2c, Additional file 1: e-Table 4).

The alanine aminotransferase to alkaline phosphatase consistently remained below 2, which is characteristic for cholestatic liver injury, in all patients with a maximal bilirubin levels during the ICU stay of > 10 μmol/l (Additional file 1: e-Figure 1). No evidence was found for a correlation between initial SARS-CoV-2 viral load at ICU admission and maximal bilirubin levels (Additional file 1: e-Figure 2).

### Time-varying weighted cumulative exposure model

The number of mechanical ventilated patients contributing data to the WCE mixed-effect model per day, as well as daily and cumulative doses of ketamine, propofol and sufentanil, is presented in Additional file 1: e-Figures 3 and 4.

**Fig. 3:**
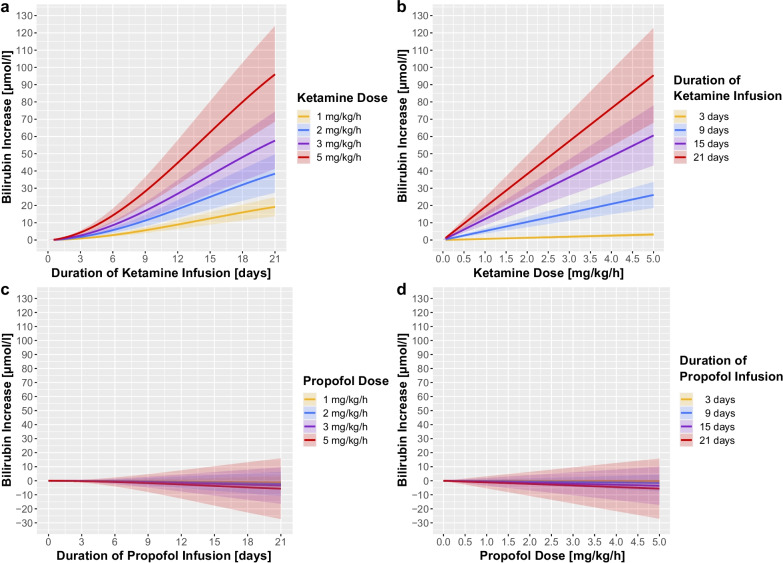
Duration–effect and dose–effect relationship between ketamine (propofol) and total bilirubin levels. Time-varying, weighted cumulative exposure mixed-effects model assessing the multivariable adjusted duration of infusion–effect (**a**) and dose–effect (**b**) relationship of ketamine (propofol (**c**, **d**)) on rising bilirubin levels. Model estimates are depicted as solid lines and 95% confidence intervals as shaded areas

The WCE mixed-effect model investigating the dose–effect relationship between ketamine infusion and daily bilirubin levels revealed a nonlinear, positively correlated, duration–effect relationship between increasing durations of ketamine infusion and rising bilirubin levels (*p* < 0.0001) (Fig. 3a, Additional file 1: e-Table 5). Furthermore, the model also showed a linear, positively correlated, dose–effect relationship between higher daily doses of ketamine and increasing bilirubin levels (*p* < 0.0001) (Fig. 3b). In comparison, long-term infusion of propofol and sufentanil, even at high doses, was not associated with increasing bilirubin levels (*p* = 0.421, *p* = 0.258) (Fig. 3c, d, Additional file 1: e-Figure 5a, 5b, Additional file 1: e-Table 5).

Other covariates associated with increasing bilirubin levels were lower age (*p* = 0.006), higher Acute Physiology and Chronic Health Disease Classification System (APACHE) II scores (*p* = 0.017), higher C-reactive protein (*p* = 0.017) levels, lower haemoglobin levels (*p* < 0.0001), higher norepinephrine doses (*p* < 0.0001), higher tidal volumes (*p* = 0.007), elevated FiO_2_ requirements (*p* = 0.013) and the use of ECMO (*p* < 0.0001) (Additional file 1: e-Table 5).

A sensitivity analysis employing the same WCE mixed-effect model, but regarding alkaline phosphatase, instead of bilirubin, as a further surrogate biomarker for cholestatic liver injury, revealed a similar strong duration–effect and dose–effect relationship with ketamine (*p* < 0.0001), which was congruently not present for propofol (*p* = 0.753) nor sufentanil (*p* = 0.818) (Additional file 1: e-Figure 5c, 5d, 6 and Additional file 1: e-Table 6). Furthermore, the effects observed in the full cohort were consistent in the sub-cohort of patients treated without ECMO (Additional file 1: e-Figures 7 and 8).

Estimated cut-offs for dose and duration of ketamine infusion leading to relevant increases in bilirubin and alkaline phosphatase levels, independently associated with ketamine, are presented in Additional file 1: e-Table 7.

### Incidence of cholestatic liver injury

**Fig. 4:**
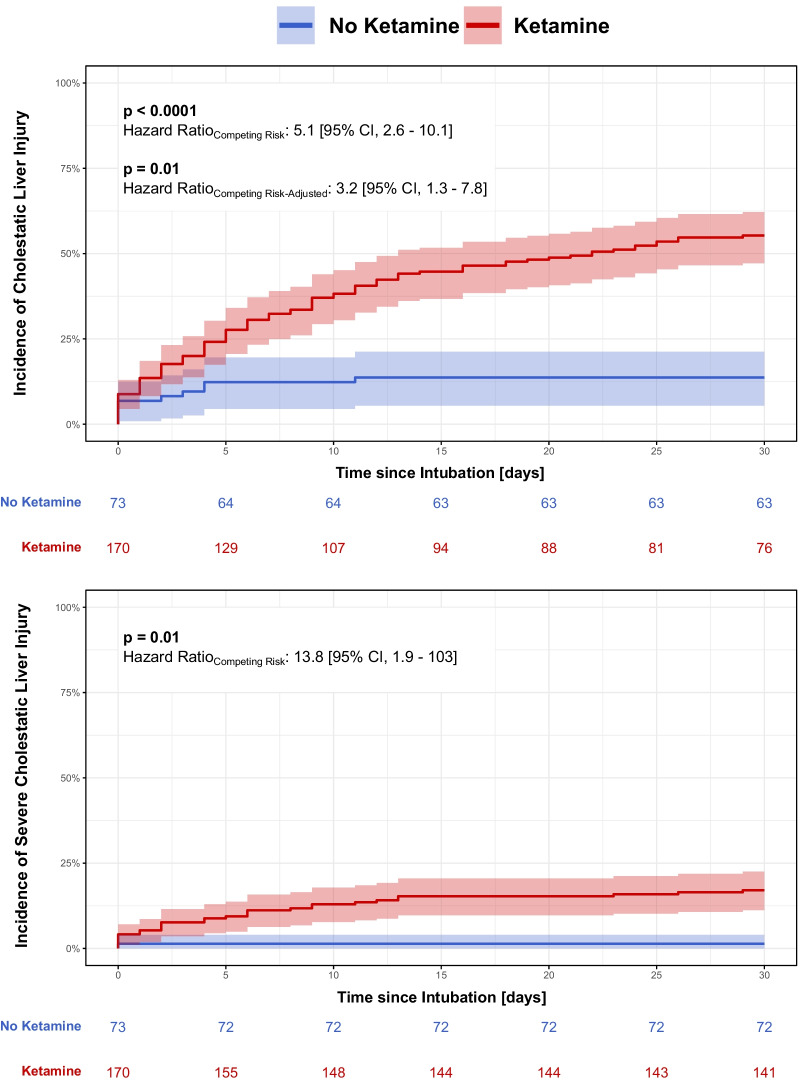
Incidence of cholestatic liver injury stratified by ketamine infusion. Kaplan–Meier curves for the 30-day incidence of **a** cholestatic liver injury and **b** severe cholestatic liver injury stratified by ketamine infusion. Shaded areas represent the crude 95% confidence intervals. The computed hazard ratio assesses the risk of ketamine-infused patients against those not having received it accounting for the competing risk of death. The 95% confidence interval is given in parentheses. Crude and multivariable adjusted hazard ratios are depicted. The underlying table presents the patients at risk per time point

Overall, 114 (47%) patients experienced a cholestatic liver injury during their ICU stay (Additional file 1: e-Table 8). Fourteen of these patients (19%) did not receive any infusion of ketamine (mild: 13, severe: 1), whereas 100 (59%) received long-term infusion of ketamine, of which 33 (33%) fulfilled the criteria for severe cholestatic liver injury. The crude competing risk hazard ratio for the incidence of cholestatic liver injury in patients infused with ketamine was 5.1 [95% confidence interval, 2.6–10.1] (*p* < 0.0001); after multivariable adjustment, the hazard rate equalled 3.2 [95% confidence interval, 1.3–7.8], *p* = 0.01) (Fig. 4a, Additional file 1: e-Figure 9, Additional file 1: e-Table 9). For the incidence of severe cholestatic liver injury, the crude competing risk hazard ratio amounted to 13.8 [95% confidence interval, 1.9–103] (*p* = 0.01) (Fig. 4b, Additional file 1: e-Figure 10).

Ketamine infusion was not associated, either in crude or in multivariable adjusted proportional hazard models, with an increased hospital mortality (Additional file 1: e-Figure 11, Additional file 1: e-Table 10).

## Discussion

The present study proposes a causal exposure–effect relationship between long-term infusion of ketamine and increased bilirubin levels in patients suffering from COVID-19-associated ARDS. This study not only portrays a dose-dependency between ketamine infusion and bilirubin increase, but also a temporal cumulative dependency between both. Furthermore, patients having received ketamine as secondary sedative agent presented substantially higher hazards of developing a cholestatic liver injury than patients not having received ketamine.

Recently, a higher-than-expected incidence of cholangiopathies, including SSCs, has been reported in patients suffering from COVID-19-associated ARDS [5, 8]. A direct SARS-CoV-2-induced damage of cholangiocytes was hypothesized as possible cause [21], as the severity of disease (in particular the severity of hemodynamic instability) did not seem to be enough to justify the increased incidence when compared to a similarly ill ARDS cohorts of different aetiology [5]. Subsequently, reports of hypothesis-generating nature suggested a putative link between the high incidence of cholangiopathies in critically ill COVID-19 and the prolonged use of ketamine in high doses as co-sedative agent in mechanically ventilated sedation-resistant COVID-19 patients [7, 22]. A similar association has recently been noted in patients with burn-induced ARDS, in which the long-term infusion of ketamine, as opposed to other sedative agents, has been linked to the inception of cholangiopathy [6].

Studies having investigated the safety of ketamine in critically ill patients have classically only considered infusion of ketamine for periods below 72 h [2, 23, 24]. These rather short exposure periods are probably not enough for ketamine to develop its deleterious effect. In fact, our analysis shows that at infusion rates of 1.5 mg/kg/h, it takes approximately 14 days until a clinically noticeable increase of 15 μmol/l in bilirubin and of 150 U/l in alkaline phosphatase, independently caused by ketamine, can be appreciated. Thus, longer periods of high-dose ketamine infusion are required to make its cholangiotoxic effect apparent, a constellation prominently given in critically ill COVID-19 patients and previously in burns patients [6]. In this line of argumentation, it has been shown that ketamine abusers, with consumption periods of over 10 years, have a unique proclivity to develop combined extrahepatic and intrahepatic cholangiopathies, which notably revert after prolonged ketamine abstinence [25].

It can of course be argued that the well-described vascular injury which characterizes critically ill COVID-19 patients [26] is highly predisposing for ischemic lesions of the biliary ducts, which are a mayor aetiology of cholangiopathy and SSC in critically ill patients [27, 28]. Most importantly, long periods of invasive ventilation with elevated fractions of inspired oxygen as well as continuous application of low tidal volumes and high positive end-expiratory pressures impair splanchnic perfusion and consecutively increase the risk of cholangiopathies [14, 29]. Long periods of mechanical ventilation are in turn associated with a higher cumulative use of sedative agents, leading to a confounding of cumulative sedative agent doses by the severity of disease. Nevertheless, the model employed in this study specifically corrects for measured confounding by severity of disease and invasiveness of organ support, considering daily changes in all possibly confounding covariates. Additionally, by entering propofol and sufentanil into the same multivariable model as ketamine, the competing effect of all three drugs on bilirubin levels could be observed. In the above-described theoretical scenario, the cumulative doses of propofol and sufentanil, being the primary sedative agents and thus even stronger correlated with the length of mechanical ventilation, would have had to have shown a greater association with bilirubin levels. The absence of such an association fortifies the here proposed causal effect of ketamine on increasing bilirubin levels and an incepting cholestatic liver injury. Moreover, the model showed the robustness of the dose–effect relationship of ketamine across two independent biomarkers of cholestatic liver injury, bilirubin and alkaline phosphatase.

Finally, considering the Bradford Hill criteria to assert causality in epidemiological studies [30], the present study was able to provide evidence on most criteria except the description of a formal molecular coupling between ketamine and cholangiotoxicity, which remains unknown (Additional file 1: e-Appendix 4). Nonetheless, different pathways have been postulated, such as a biliary tract dysfunction, accumulation of bile and dilation of the bile ducts induced by a blockade of the NMDA receptor in smooth muscle cells through ketamine [31]. The contraction of the sphincter of Oddi, in response to ketamine, inducing an increase in flow resistance could possibly further exacerbate this bile accumulation [25, 32]. Additionally, by blockade of NMDA receptors in the dorsal motor nucleus of the vagal nerve, gall bladder dyskinesia could be induced [33]. These multiple pathways leading to bile stasis might then conjointly predispose for the precipitation of norketamine, the mayor active metabolite of ketamine, into the bile, which would then lead to biliary obstruction, cholangitis and finally secondary biliary cirrhosis [7]. These pathways could all be aggravated by progressing renal failure and an inability to clear ketamine and its metabolites.

The present analysis has limitations. *First*, the present study was not a randomized controlled trial; thus, the possibility for unobserved confounding to be present cannot be excluded. However, due to the prospective collection of the underlying data, with a minimal missing rate, coupled to the pre-specified causal pathway and the implementation of a time-varying, mixed-effects model, at least observed confounding could be maximally mitigated. *Second*, this is an analysis of a single-centre prospective observational study and generalizability of the results is not given. Conversely, our analysis is supported by a number of studies that have strengthened the plausibility of a causal injurious effect of long-term ketamine infusion. *Third*, patients requiring longer periods of mechanical ventilation and subsequently higher cumulative doses of second-line sedative agents, such as ketamine, were presumably more severely ill and at a higher risk of developing accompanying cholestatic liver injury. Nevertheless, the demonstrated absence of a comparable effect of propofol and sufentanil in the very same patient cohort supports our proposed dose–response effect of ketamine on bilirubin and alkaline phosphatase levels. *Finally*, by design, this study could not show a direct causal effect of ketamine on histological cholangiocyte damage, preventing any causal inference on the progression from cholestatic liver injury to cholangiopathy.

## Conclusion

In conclusion, we present a causally plausible, dose–effect relationship between long-term ketamine infusion and an increase in total bilirubin levels as well as an augmented hazard of cholestatic liver injury in patients suffering from COVID-19-associated ARDS. Considering the growing body of evidence, we recommend against the use of high-dose ketamine as long-term analgosedative agent in mechanically ventilated COVID-19 patients. If, however, faced with the necessity to employ ketamine, bilirubin and alkaline phosphatase should be monitored daily and ketamine infusion should be halted when systematic elevations become apparent, usually after 14 days.

## Supplementary Information

**Additional file 1**. Supplementary information.

## Data Availability

All data analysed and discussed in the framework of this study are included in this published article and its online supplementary information. The corresponding author may provide specified analyses or fully de-identified parts of the data set upon reasonable request.

## Abbreviations

APACHE: Acute Physiology and Chronic Health Disease Classification System
ARDS: Acute respiratory distress syndrome
BMI: Body mass index
COVID-19: Coronavirus disease 2019
ECMO: Extracorporeal membrane oxygenation
FiO_2_: Inspired oxygen fraction
ICU: Intensive care unit
NMDA: N-methyl-D-aspartate
P/F ratio: Partial pressure of arterial oxygen over inspired oxygen fraction ratio
paO_2_: Partial pressure of arterial oxygen
SSC: Secondary sclerosing cholangitis
STROBE: Strengthening the Reporting of Observational Studies in Epidemiology
WCE: Weighted cumulative exposure
