# Supplementary material for: Long-term ketamine infusion-induced cholestatic liver injury in COVID-19-associated acute respiratory distress syndrome
Source: Crit Care. 2022 May 23;26:148. doi: 10.1186/s13054-022-04019-8 (PMC9125956; doi:10.1186/s13054-022-04019-8)
Supplement: Supplementary file 1 — Additional file 1. Supplementary information. [file 13054_2022_4019_MOESM1_ESM.pdf]

Supplementary Material

Long-term ketamine infusion induced cholestatic liver injury in COVID-19 associated acute respiratory distress syndrome

Wendel-Garcia PD, Erlebach R, Hofmaenner DA, Camen G, Schuepbach RA, Jüngst C, Müllhaupt B, Bartussek J, Buehler PK, Andermatt R, David S

Table of contents

e-Table 1. Sedation requirements in critically ill, non-COVID and COVID ARDS patients 4

e-Table 2. Missing Data 7

e-Appendix 1. Directed acyclic graph for time-indpendent causal model 8

e-Appendix 2. Directed acyclic graph for time-varying causal model 9

e-Appendix 3. Cumulative drug exposure model 10

e-Table 3. Association between total ketamine dose and maximal circulating bilirubin – Univariable cubic regression B-spline 13

e-Table 4. Association between total ketamine dose and maximal circulating bilirubin – Multivariable cubic regression B-spline 14

e-Figure 1. Alanine Aminotransferase/ Alkaline Phosphatase Ratio 15

e-Figure 2. Correlation between max. bilirubin levels and initial SARS-CoV-2 viral load 16

e-Figure 3. Deaths, number of mechanically ventilated contributing data as well as daily and cumulative ketamine doses 17

e-Figure 4. Daily and cumulative propofol and sufentanil doses 18

e-Figure 5. Duration- and dose-effect relationship between sufentanil and bilirubin/ alkaline phosphatase levels 19

e-Table 5. Time-varying weighted cumulative exposure mixed-effects model for total bilirubin 20

e-Figure 6. Duration- and dose-effect relationship between ketamine (propofol) and alkaline phosphatase levels 21

e-Table 6. Time-varying weighted cumulative exposure mixed-effects model for alkaline phosphatase 22

e-Figure 7. Duration- and dose-effect relationship between ketamine (propofol) and total bilirubin levels in mechanically ventilated patients without ECMO 23

e-Figure 8. Duration- and dose-effect relationship between ketamine (propofol) and alkaline phosphatase levels in mechanically ventilated patients without ECMO 24

e-Table 7. Cut-offs for average daily infusion rate and duration of ketamine infusion for different increases in bilirubin and alkaline phosphatase levels.............25

e-Table 8. Incidence of cholestatic liver injury, organ support and outcomes 26

e-Figure 9. Cumulative incidence functions for cholestatic liver injury and death 27

e-Table 9. Multivariable Fine and Gray competing risk proportional hazards model for the incidence of cholestatic liver injury accounting for death 28

e-Figure 10. Cumulative incidence functions for severe cholestatic liver injury and death 29

e-Figure 11. Kaplan-Meier and Cox proportional hazards model for hospital mortality 30

e-Table 10. Multivariable Cox proportional hazards model for hospital survival....................31

e-Appendix 4. Bradford Hill Criteria for causal inference in epidemiological association studies 32

References 33

e-Table 1. Sedation requirements in critically ill, non-COVID and COVID ARDS patients

Title	Patients	Findings	Number of patients	Evidence type	Year	Citation
Clinical Practice Guidelines for the Prevention and Management of Pain, Agitation/Sedation, Delirium, Immobility, and Sleep Disruption in Adult Patients in the ICU	Critically Ill	*"Question: Should ketamine be used as an adjunct to an opioid (vs an opioid alone) for pain management in critically ill adults?"* *"Recommendation: We suggest using low-dose ketamine (0.5 mg/kg IVP x 1 followed by 1-2 µg/kg/min infusion) as an adjunct to opioid therapy when seeking to reduce opioid consumption in postsurgical adults admitted to the ICU (conditional recommendation, very low quality of evidence)."*		Guidelines	2018	Crit Care Med. 2018 Sep;46(9):e825-e873
Sedation of Mechanically Ventilated COVID-19 Patients: Challenges and Special Considerations	COVID ARDS	*"Unusually high sedation requirements in alarge proportion of COVID-19 patients are observed in current clinical experience."*		Commentary	2020	Anesth Analg. 2020 Jul;131(1):e40-e41.
The Use of Analgesia and Sedation in Mechanically Ventilated Patients With COVID-19 ARDS	COVID ARDS	*"High analgesic and sedative medication requirements were observed in a cohort of patients with COVID-19–related ARDS, with doses exceeding those previously documented in the literature for patients with ARDS"*	N = 24	Retrospective, single center (John Hopkins Hospital)	2020	Anesth Analg. 2020 Oct;131(4):e198-e200.

Analgesia and sedation in patients with ARDS	ARDS (Non-COVID and COVID)	*"Early experiences in the COVID-19 pandemic have seen changes in the approach to sedation, with a tendency towards deep sedation and a resurgence of the use of benzodiazepine infusions."*<br>Explicitly mentioned second-line sedative at doses 1-3mg/kg/h, in order to achieve deep sedation for COVID-19. The only adverse events mentioned at high doses (>1mg/kg/h) are emerging hallucinations and decreased cardiac output.		Review	2020	Intensive Care Med. 2020 Dec;46(12):2342-2356.
High sedation needs of critically ill COVID-19 ARDS patients—A monocentric observational study	COVID ARDS	Triple sedation regimen with clonidine, esketamine and midazolam in 75.7% to achieve prescribed sedation level.	N = 56	Retrospective, Single center experience	2021	PLoS One. 2021 Jul 27;16(7):e0253778.
An examination of sedation requirements and practices for mechanically ventilated critically ill patients with COVID-19	COVID, invasive ventilation	The doses of sedatives increased over the first 10 days, reaching or exceeding the upper limits of dosage guidelines for propofol (48% of pat.), dexmedetomidine (29%), midazolam (7.7%), ketamine (32%), and hydromorphone (38%). More than 50% of patients required 3 or more agents by day 2.	N = 86	Retrospective,<br>8 ICUs, Single center (MGH, Boston)	2021	Am J Health Syst Pharm. 2021 Oct 25;78(21):1952-1961.
Association of Sedation, Coma, and In-Hospital Mortality in Mechanically Ventilated Patients With Coronavirus Disease 2019-Related Acute	COVID ARDS<br>vs.	Sedation target RAS ≤ -3, ketamine was used in 51.8% of COVID but only in 0.9% of non-COVID ARDS.<br>Ketamine dose: 919 (610–1'570) mg/d	N = 114<br>vs.	Propensity matched	2021	Crit Care Med. 2021 Sep 1;49(9):1524-1534.

Respiratory Distress Syndrome: A Retrospective Cohort Study	Non-COVID ARDS		N = 228	cohort study, retrospective		
Challenges in the management of analgesia and sedation in critically ill patients with COVID-19 in Chile	COVID ARDS	Algorithm for the sedation of COVID-19 specifically recommending the use of ketamine		Review	2021	Rev Med Chil. 2021 Apr;149(4):559-569
Impact of ketamine as an adjunct sedative in acute respiratory distress syndrome due to COVID-19 Pneumonia	COVID ARDS	Propofol and norepinephrine sparing effect at 72 h after ketamine initiation when compared to 24 h (median 34.2 vs 54.7 mg/kg, p = 0.003).	N = 59	Observational retrospective study	2021	Respir Med. Nov-Dec 2021;189:106667.
Challenges in sedation management in critically ill patients with COVID-19: a Brief Review	COVID ARDS	*"These patients tend to require higher doses of sedative medications and often for long periods of time.*"		Review	2021	Curr Anesthesiol Rep . 2021 Feb 26;1-9.
A Dual-Center Cohort Study on The Association Between Early Deep Sedation and Clinical Outcomes in Mechanically Ventilated Patients During the COVID-19 Pandemic: the COVID-SED Study	COVID ARDS	Comparison between deep and light early sedation. Overall 12.7% of all patients were on ketamine.	N = 391	Dual-center retrospective cohort study	2022	Res Sq. 2022 Mar 1;rs.3.rs-1389892.

e-Table 2. Missing Data

	Missing [%]
Total time-points [days]	*4150*
Acute Physiology and Chronic Health disease Classification System II – *APACHE II Score*	0
Age	0
Alkaline Phosphatase	2.8
Arterial partial pressure of oxygen – *paO2*	1.2
Bilirubin	0.6
Central venous pressure	1.9
C-reactive protein – *CRP*	4.3
Creatinine	4.6
Extracorporeal membrane oxygenation – *ECMO*	0
Haemoglobin	4.4
Inspiratory oxygen fraction – FiO_2	0
Ketamine dose	0
Norepinephrine dose	0
Positive end-expiratory pressure – *PEEP*	0.5
Procalcitonin – *PCT*	12.5
Prone position	0
Propofol dose	0
Sex	0
Systolic arterial pressure	0.1
Tidal volume	4

e-Appendix 1. Directed acyclic graph for time-indpendent causal model

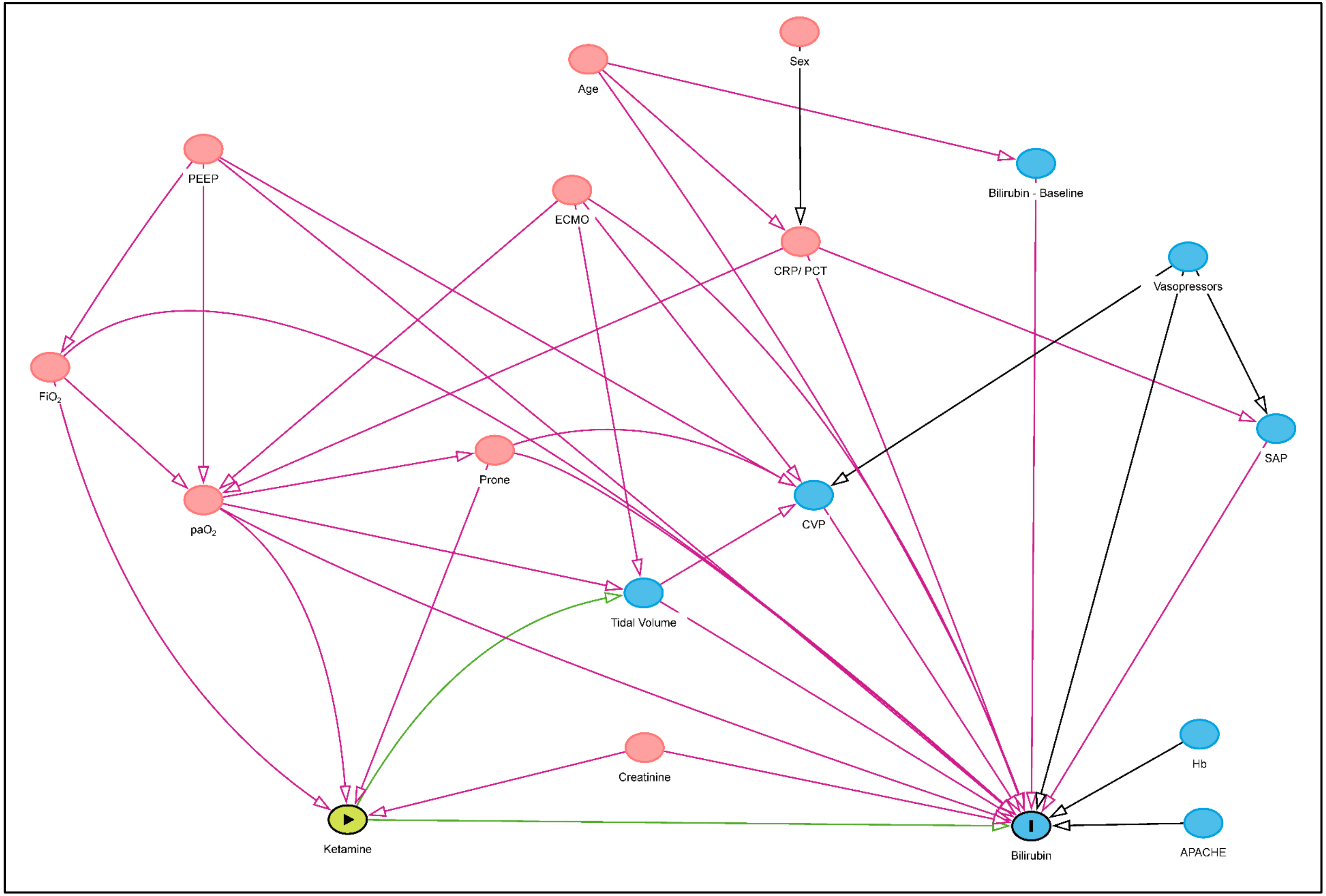

Directed acyclic graph depicting the conceptual model of causal association between ketamine and bilirubin. Ketamine (exposure) and its directed ancestors are coloured in green, whereas bilirubin (outcome) and its direct ancestors are coloured in blue. Ancestors of both ketamine and bilirubin are coloured in pink. Arrows present a directed causal and hierarchical relationship between two variables.

<u>Abbreviations:</u> APACHE – Acute Physiology and Chronic Health disease Classification System; CRP – C-reactive protein; CVP – Central venous pressure; ECMO – Extracorporeal membrane oxygenation; FiO_2 – Fraction of inspired oxygen; Hb –Haemoglobin; paO_2 – partial pressure of arterial oxygen; PCT – Procalcitonin; PEEP – Positive end-expiratory pressure; SAP – Systolic arterial pressure.

e-Appendix 2. Directed acyclic graph for time-varying causal model

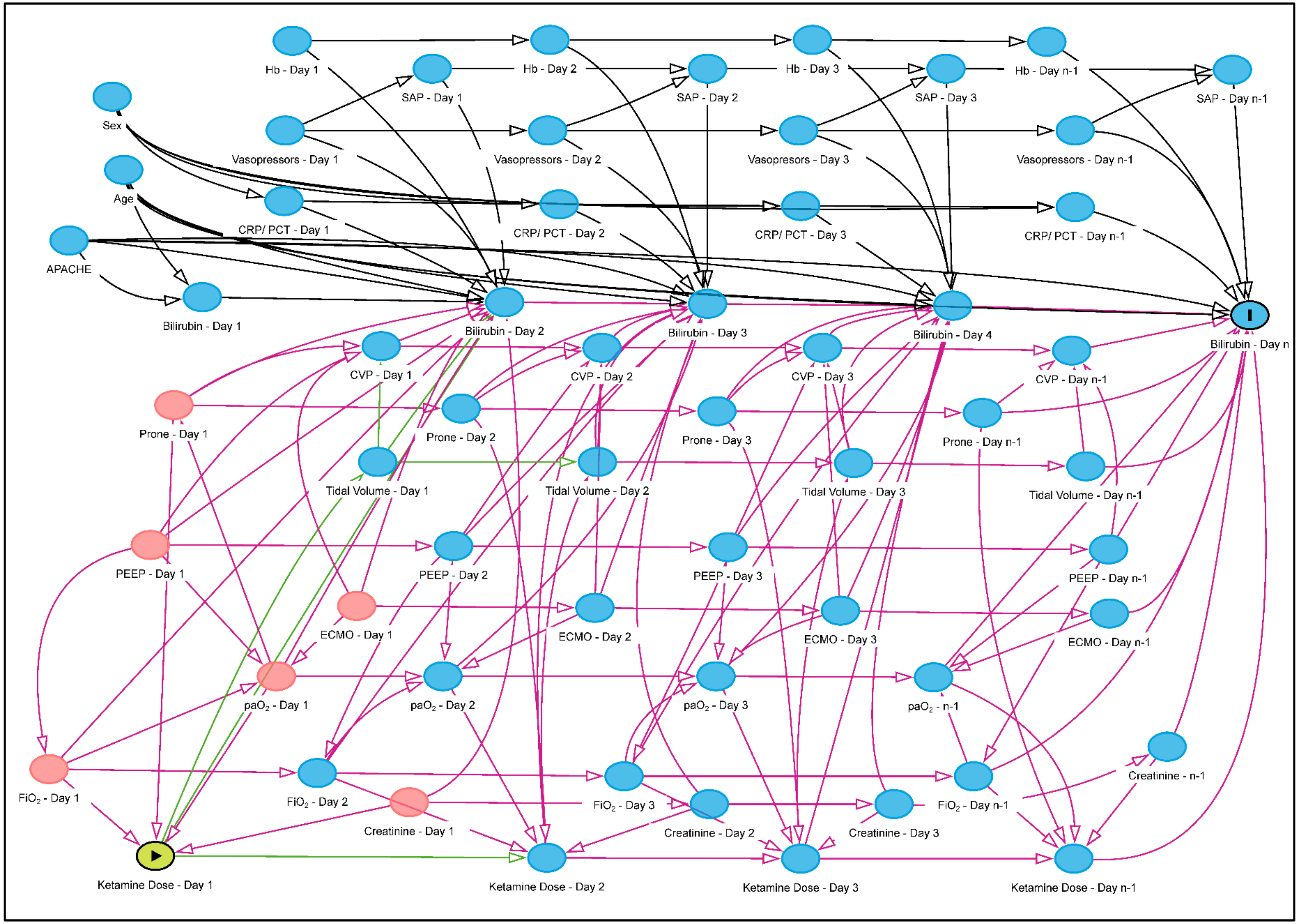

Longitudinal directed acyclic graph depicting the conceptual time-varying model of causal association between continuous daily ketamine infusion (exposure) and daily circulating bilirubin (outcome). Arrows present a directed causal and hierarchical relationship between two variables.

<u>Abbreviations:</u> APACHE – Acute Physiology and Chronic Health disease Classification System; CRP – C-reactive protein; CVP – Central venous pressure; ECMO – Extracorporeal membrane oxygenation; FiO_2 – Fraction of inspired oxygen; Hb – Haemoglobin; paO_2 – partial pressure of arterial oxygen; PCT – Procalcitonin; PEEP – Positive end-expiratory pressure; SAP – Systolic arterial pressure.

e-Appendix 3. Cumulative drug exposure model

Exposure-response studies represent the sole analytical methodology to explore the risk associated with exposition to a substance. Generally anaesthetic agents are evaluated in the time-frame of hours to days, enabling classic pharmacokinetic and pharmacodynamic studies to be performed. However, the impact of a drug infused over a long period of time (multiple days to weeks) at a distinct time-point can usually not be described by the isolated dose applied at said single time-point, as the effect generally cumulates over time [1]. It is important to note that knowledge about short-term pharmacodynamics, including context sensitive half-life, is usually not sufficient to understand how a drug behaves in long-term infusion settings [1-3]. Additionally, the relative effect of past drug doses is affected by the time-point of exposure [2, 4]. The weighted cumulative exposure (WCE) approach models the effect of a drug as the weighted sum of all past exposures, were *u* represents the time of assessment, *t* the time when the exposure originated and *X(t)* the effective dose at *t* [2, 5]:

$$WCE\ (u) = \sum_{t \leq u} w(u-t) \times X(t)$$

The complexity of the weighting function can be adapted from simple linear relationships to more complex polynomials estimated based on simulation studies [5, 6]. For the purpose of this study we chose an exponential weight function as employed in similar studies exploring the effects of the long-term application of benzodiazepines, with σ representing the rate of change per unit time [2]:

$$w(u-t) = e^{-\frac{(u-t)^2}{\sigma^2}}$$

In order to define the most appropriate σ for the weight function, the complete final mixed-effect model was generated for different σ. The weight function characterized by a σ that lead to a 0.5 weight after 12 days was selected for ketamine and propofol (4 days for sufentanil) as it minimized akaike's information criterion (AIC) [2, 4].

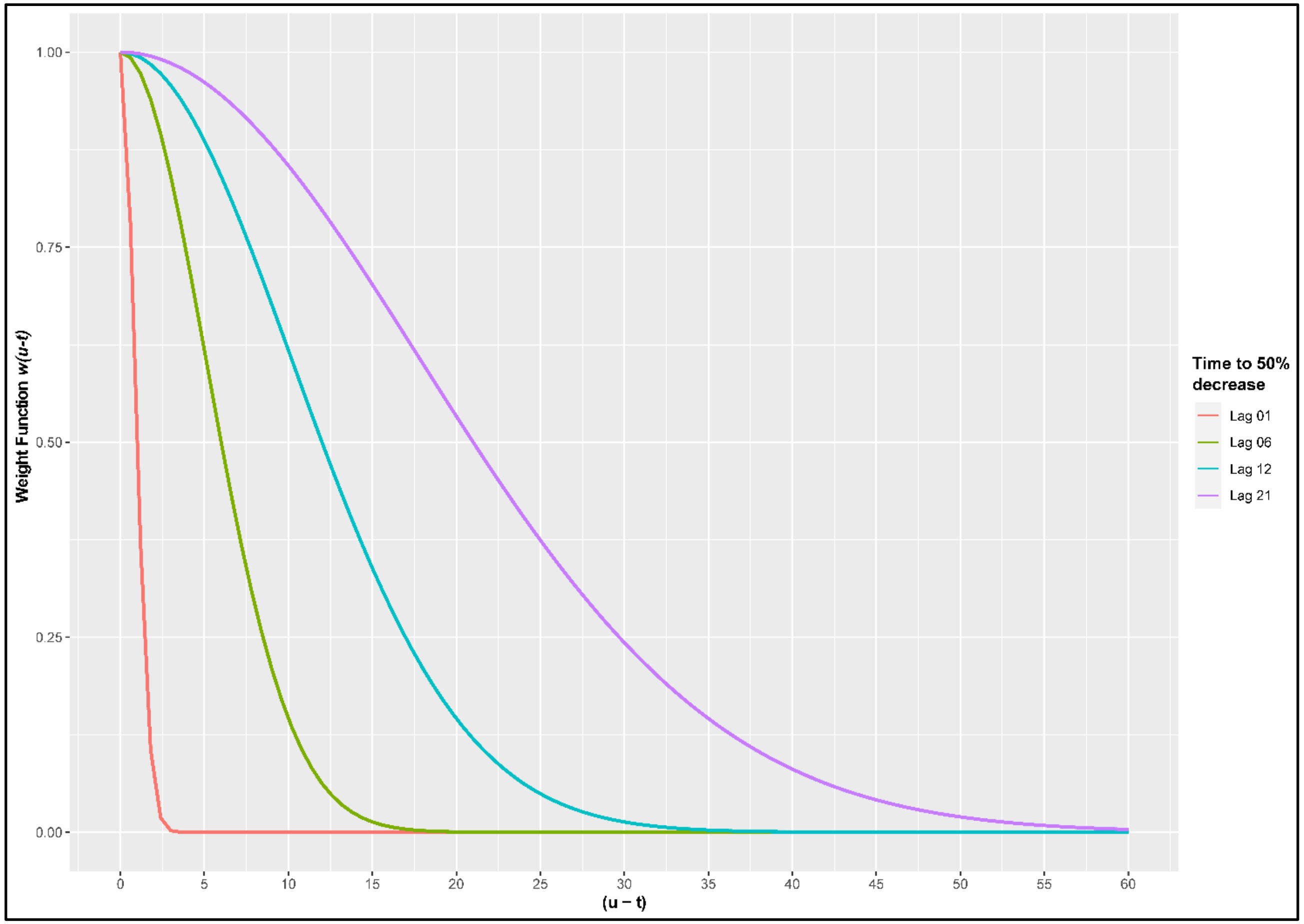

Weight functions based on an exponential function modelling the relative effect of a past drug exposure as a function of the time elapsed between the assessment time-point u and a point t in the past. Depending on the lag employed, the function takes longer or shorter time to decay to 50% of the original effect.

To date most exposure-response studies have been proposed as binary-outcome or time-to-event analyses, until very recently no methodologies had been proposed to study the time-varying effect of an exposure on a repeated measures outcome [4]. However, especially in the highly volatile setting of intensive care units, were

exposure-effect relationships can easily be confounded by a parallel derangement of the patient's status, exposure-response models able to account not only for time-varying exposures and outcomes, but also to time-varying covariates are required to enable a causal assessment. Recently, the group around Michal Abrahamowicz, who was the initial proposer of the WCE approach, have demonstrated how the weight function can be incorporated into a linear effects mixed model, with Y representing the repeated time-varying outcome, i the time-point of evaluation for patient k, β_0 the overall intercept for all patients at time-point 0, $b_{i,0}$ the random intercept for patient i, β_{WCE} the fixed effect associated with a change in Y for every unit increase in WCE, β_s the fixed effect for the covariate Cov_s and ε_{ik} the random slope effect accounting for temporal correlations among repeated measures from the same patient, as previously described [4]:

$$Y_i(t_{ik}) = \beta_0 + b_{i,0} + \beta_{WCE}\, WCE_i(t_{ik}) + \sum_{s=1}^{n_{Cov}} (\beta_s Cov_{s,ik}) + \varepsilon_{ik}$$

We employed the proposed mixed-effects model including three WCE terms, one for ketamine, one for propofol and one for sufentanil, and all covariates previously defined in the causal model described in e-Appendix 2. We included ketamine, propofol and sufentanil into the model in order to provide direct comparators to the investigated causal exposure effect of ketamine on bilirubin. Propofol and sufentanil were chosen as they were continuously infused over the whole period of mechanical ventilation as opposed to ketamine, which was only employed during the periods of deepest sedation. In order to allow a robust quantification of the daily vasopressor requirement of the patient, the daily cumulative norepinephrine dose was employed in the model.

e-Table 3. Association between total ketamine dose and maximal circulating bilirubin – Univariable cubic regression B-spline

	Estimate	Standard Error	p
**Fixed Effects**			
Intercept	11.455	5.888	0.052
**Ketamine Deciles – B-Spline 1**	-6.949	8.860	0.433
**Ketamine Deciles – B-Spline 2**	56.320	14.976	<0.001
**Ketamine Deciles – B-Spline 3**	52.269	6.488	<0.0001

e-Table 4. Association between total ketamine dose and maximal circulating bilirubin – Multivariable cubic regression B-spline

	Estimate	Standard Error	p
**Fixed Effects**			
Intercept	8.8646465	39.7914777	0.823810
**Ketamine Deciles – B-Spline 1**	15.001	9.999	0.134
**Ketamine Deciles – B-Spline 2**	14.399	16.876	0.394
**Ketamine Deciles – B-Spline 3**	27.015	8.049	<0.001
**Age, years**	-0.492	0.302	0.105
**Sex, Male**	9.078	7.728	0.242
**Baseline bilirubin, µmol/l**	1.742	0.144	<0.0001
**Norepinephrine dose, µg/kg/min**	0.435	0.087	<0.0001
**Haemoglobin, g/l**	-0.295	0.208	0.157
**Systolic arterial pressure, mmHg**	-0.029	0.327	0.929
**Central venous pressure, mmHg**	-0.169	0.213	0.429
**paO_2, kPa**	-1.852	3.527	0.600
**FiO_2, %**	0.212	0.190	0.266
**APACHE II Score**	0.481	0.533	0.368
**Prone position, yes**	7.445	8.581	0.387
**Tidal volume, ml/kg**	3.413	1.837	0.065
**PEEP, cmH_2O**	-1.307	1.250	0.297
**C-reactive protein, mg/l**	0.068	0.033	0.039
**Procalcitonin, µg/l**	0.008	0.090	0.926
**ECMO, yes**	25.580	9.895	0.010

The worst value during the intensive care unit stay was employed for every covariate.

Abbreviations: APACHE – Acute Physiology and Chronic Health disease Classification System; ECMO – Extracorporeal membrane oxygenation; FiO_2 – Fraction of inspired oxygen; paO_2 – partial pressure of arterial oxygen; PEEP – Positive end-expiratory pressure.

e-Figure 1. Alanine Aminotransferase/ Alkaline Phosphatase Ratio

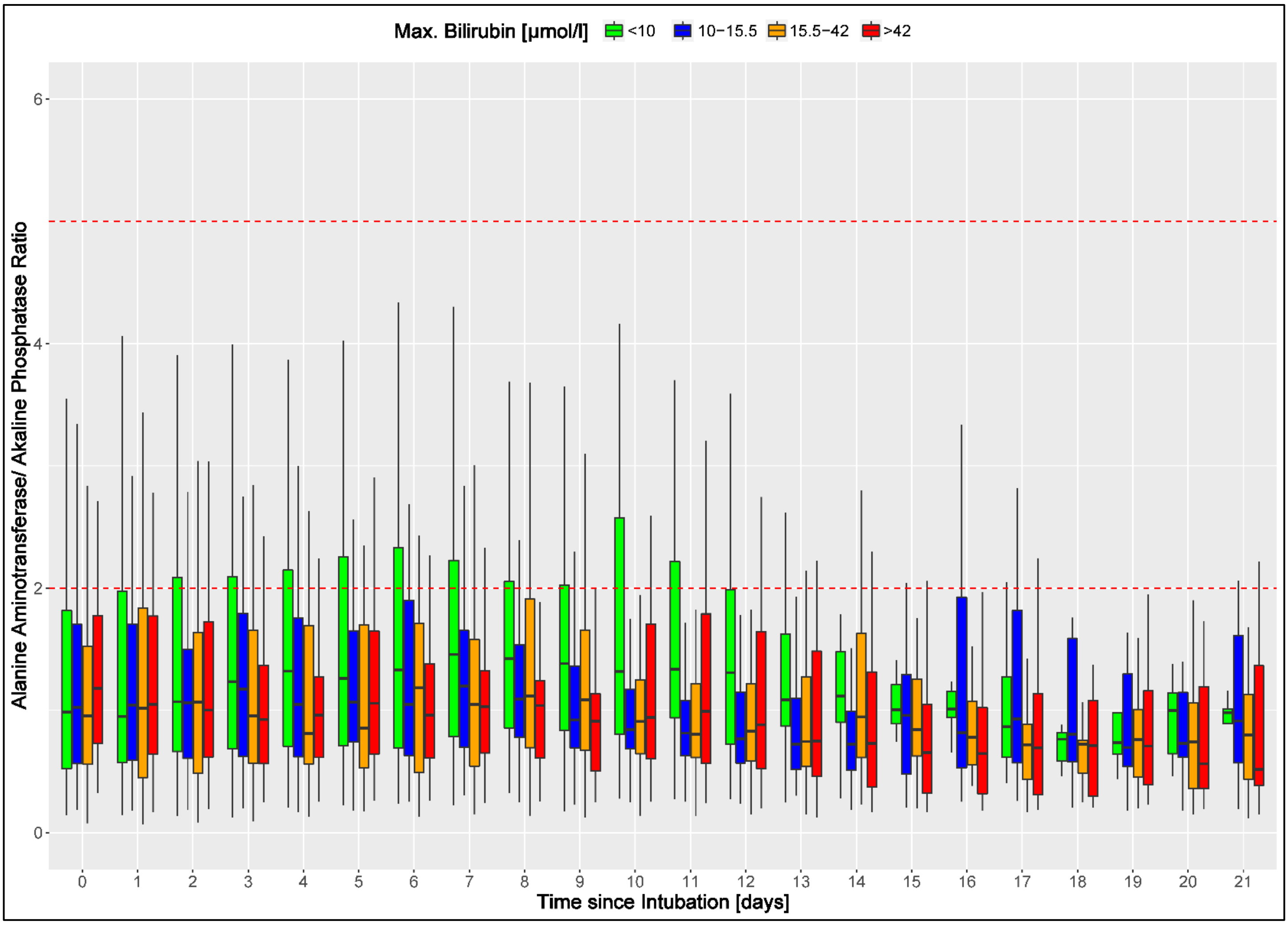

Boxplots stratified by maximal bilirubin levels during the intensive care unit stay depicting the progression of the ratio of alanine aminotransferase to alkaline phosphatase (both normalized to their respective upper limit of normality). A ratio > 5 is indicative of a hepatocellular liver injury, whereas a ratio < 2 is characteristic for a cholestatic liver injury.

e-Figure 2. Correlation between max. bilirubin levels and initial SARS-CoV-2 viral load

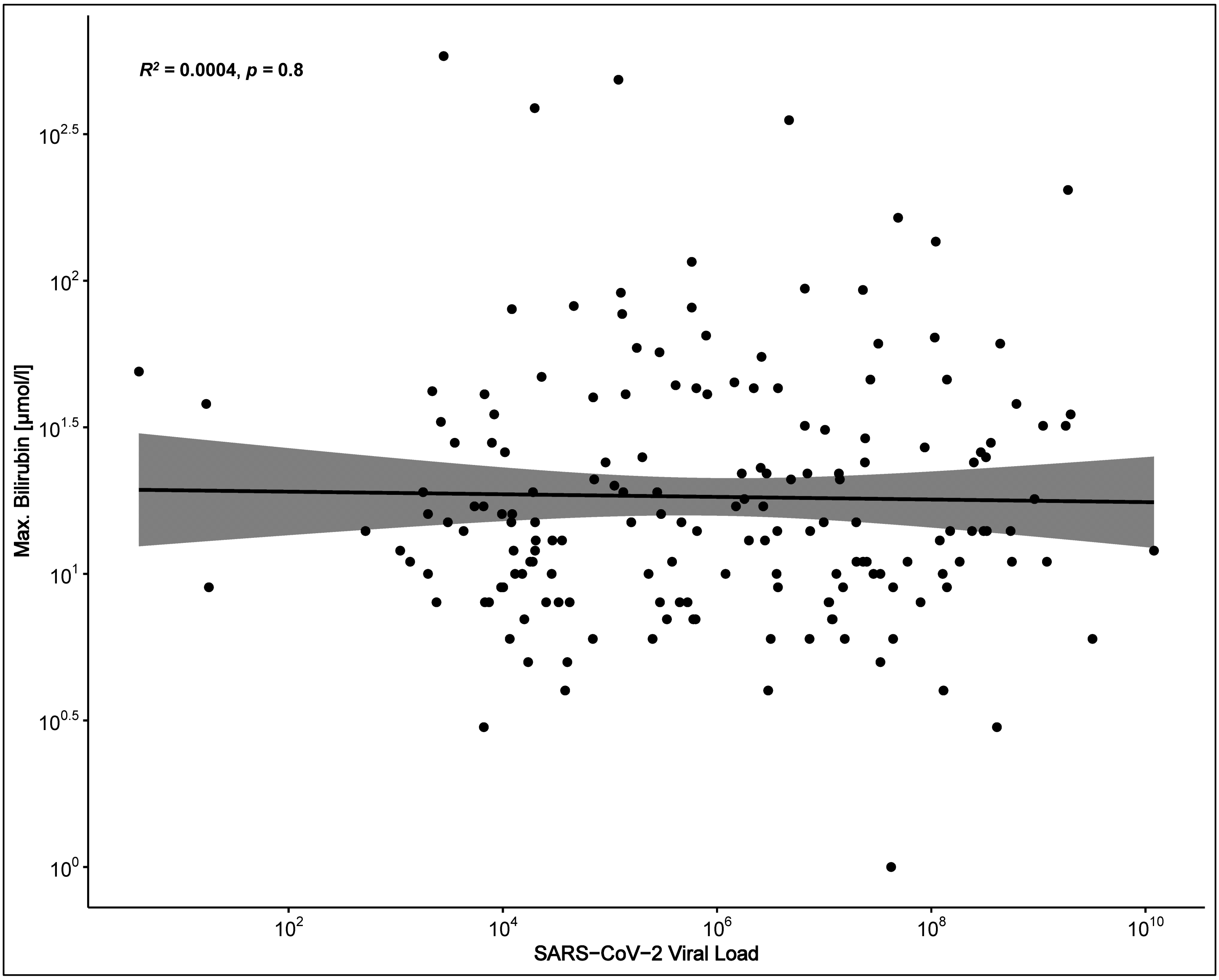

Correlation plot between maximal bilirubin levels during the intensive care unit stay and SARS-CoV-2 viral load at intensive care unit admission.

e-Figure 3. Deaths, number of mechanically ventilated contributing data as well as daily and cumulative ketamine doses

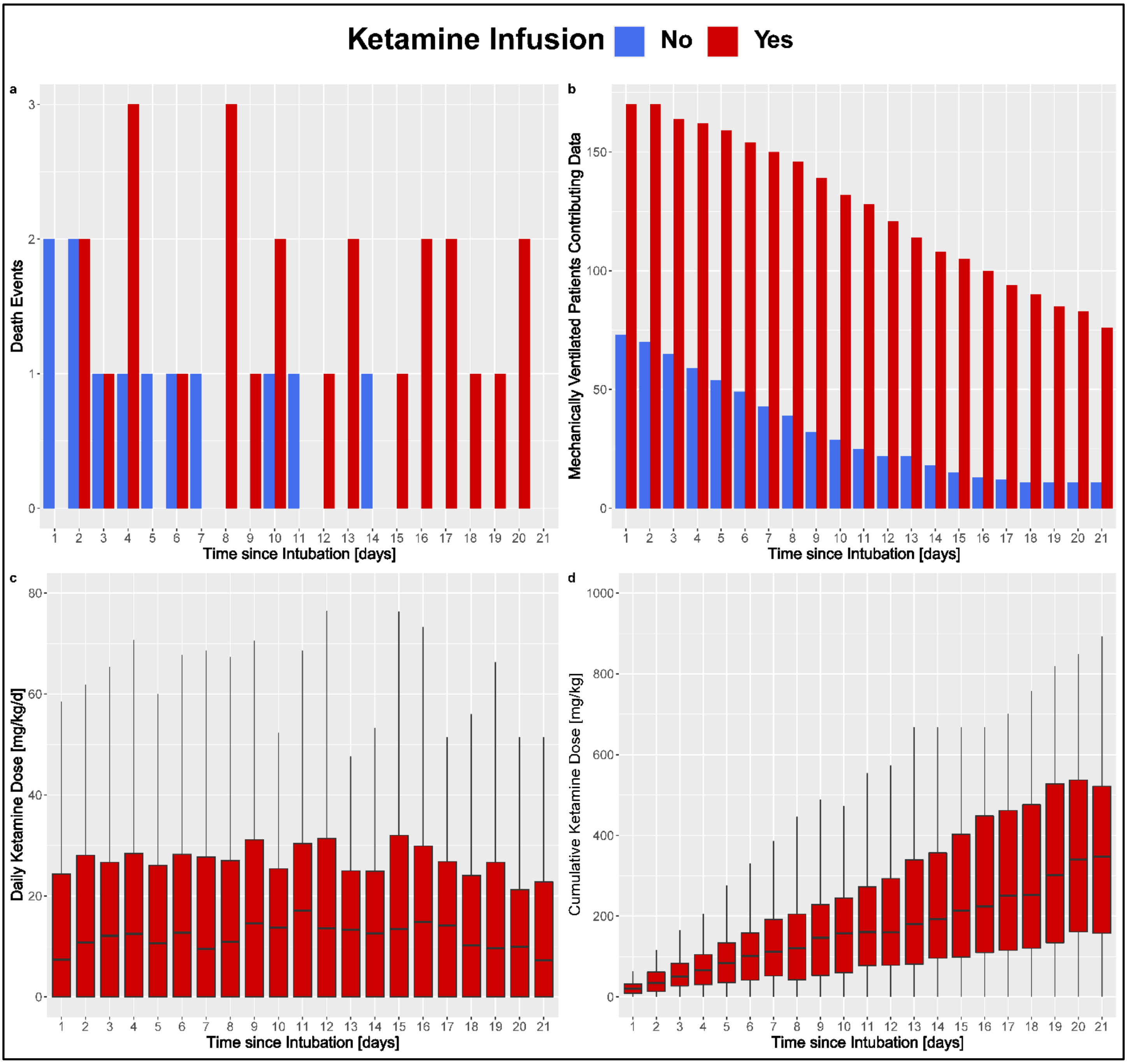

Bar plots depicting **(a)** death events and **(b)** the number of mechanically ventilated patients contributing data to the time-varying weighted cumulative exposure model stratified by ketamine infusion over time. Boxplots presenting the distribution of **(c)** daily and **(d)** cumulative ketamine doses in mechanically ventilated patients stratified by ketamine infusion over time.

e-Figure 4. Daily and cumulative propofol and sufentanil doses

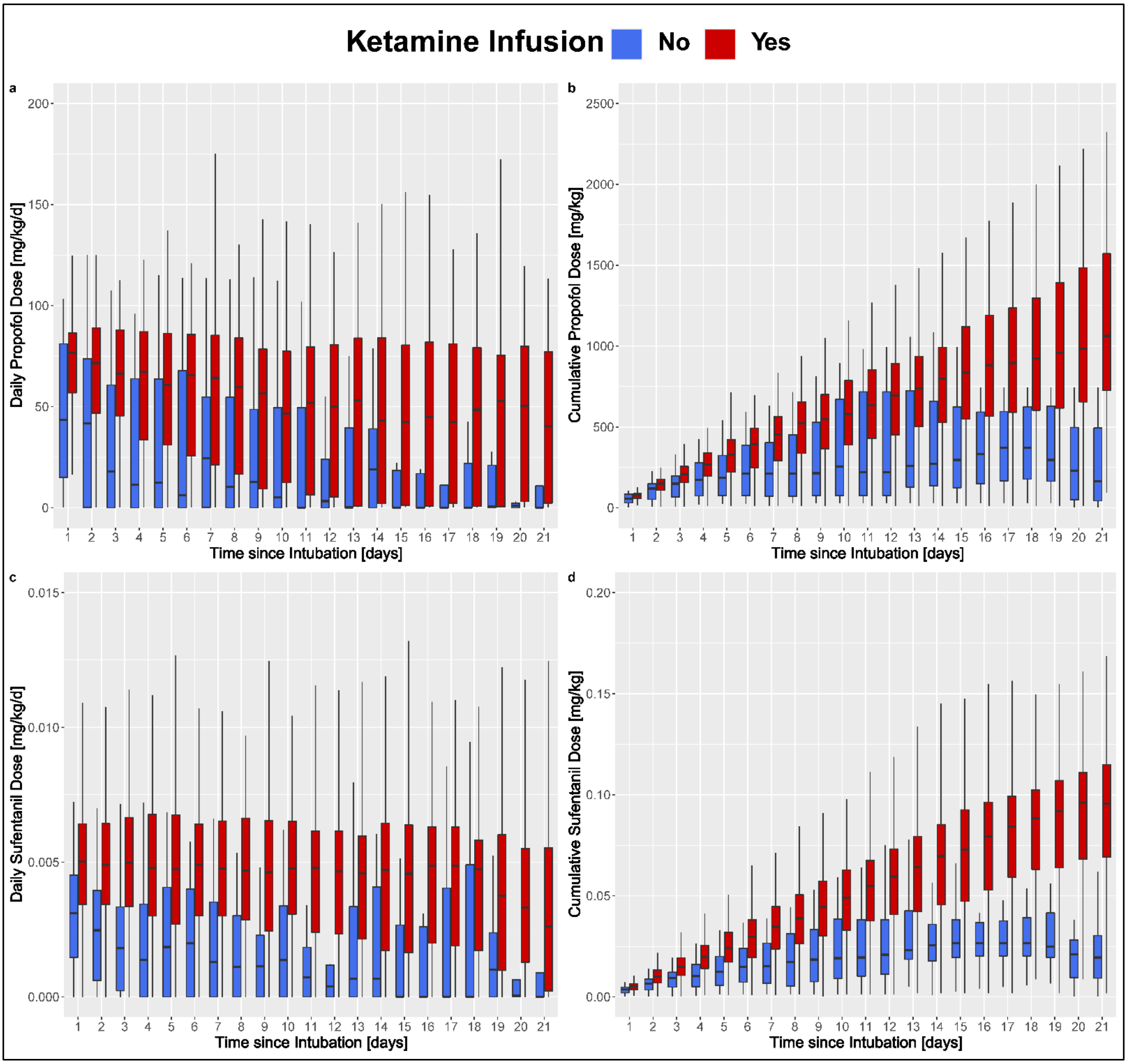

Boxplots presenting the distribution of **(a, c)** daily and **(b, d)** cumulative propofol as well as sufentanil doses in mechanically ventilated patients stratified by ketamine infusion over time.

e-Figure 5. Duration- and dose-effect relationship between sufentanil and bilirubin/ alkaline phosphatase levels

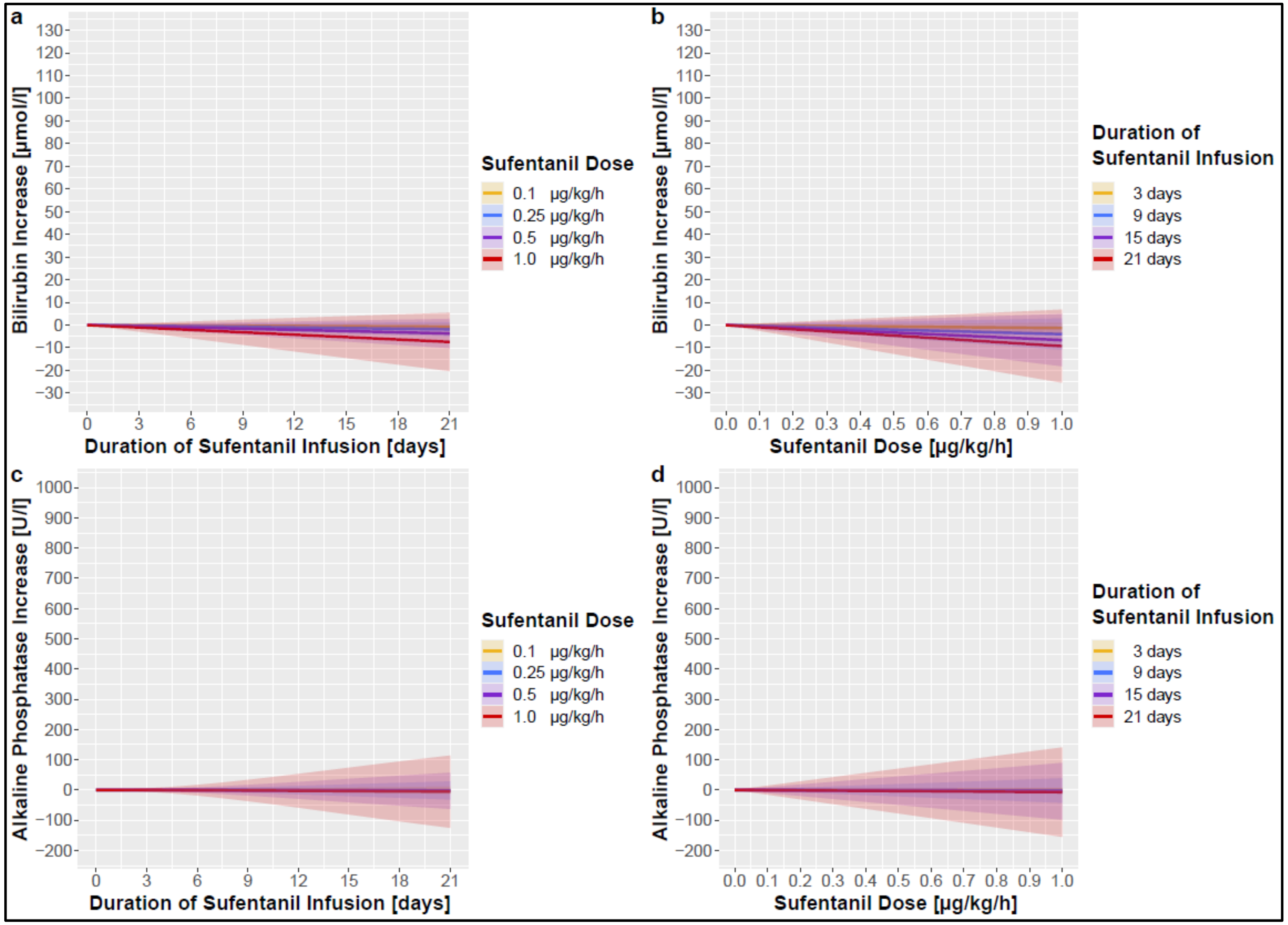

Time-varying, weighted cumulative exposure mixed-effects model assessing the multivariable adjusted duration of infusion-effect **(a, c)** and dose effect **(b, d)** relationship of sufentanil on rising bilirubin **(a, b)** and alkaline phosphatase **(c, d)** levels. Model estimates are depicted as solid lines and 95% confidence intervals as shaded areas.

e-Table 5. Time-varying weighted cumulative exposure mixed-effects model for total bilirubin

	Variance		Standard Deviation	
**Random Effect**				
Intercept	1742.593		41.74	
Slope	4.928		2.22	
	**Interaction**	**Estimate**	**Standard Error**	**p**
**Fixed Effects**				
Intercept	Static	1.426e+01	1.912e+01	0.456
**Ketamine, mg/kg/d**	WCE	2.980e-03	4.372e-04	<0.0001
**Propofol, mg/kg/d**	WCE	-2.751e-03	3.415e-04	0.421
**Sufentanil, µg/kg/d**	WCE	-1.845e+01	1.630e+01	0.258
**Age, years**	Static	-6.839e-01	2.483e-01	0.006
**APACHE II Score**	Static	1.195e+00	4.957e-01	0.017
**Central venous pressure, mmHg**	Static	6.978e-03	2.415e-02	0.773
	Time-Dependent	-8.016e-05	2.178e-03	0.971
**C-reactive protein, mg/l**	Static	1.904e-02	8.004e-03	0.017
	Time-Dependent	4.479e-04	8.402e-04	0.594
**Creatinine, µmol/l**	Static	2.295e-02	1.308e-02	0.079
	Time-Dependent	-3.590e-03	1.512e-03	0.018
**ECMO, yes**	Static	1.621e+01	3.331e+00	<0.0001
	Time-Dependent	-1.806e+00	2.857e-01	<0.0001
**FiO_2, %**	Static	1.063e-01	4.276e-02	0.013
	Time-Dependent	1.133e-02	4.693e-03	0.016
**Haemoglobin, g/l**	Static	-2.534e-01	5.311e-02	<0.0001
	Time-Dependent	-1.754e-02	1.512e-03	0.0176
**Norepinephrine dose, mg/kg/d**	Static	3.037e+01	5.724e+00	<0.0001
	Time-Dependent	1.179e+00	5.842e+00	0.044
**paO_2, kPa**	Static	-2.449e-01	3.438-01	0.476
	Time-Dependent	1.926e-03	2.819e-02	0.946
**PEEP, cmH_2O**	Static	-6.599e-01	2.908e-01	0.023
	Time-Dependent	2.373e-02	2.801e-02	0.397
**Procalcitonin, µg/l**	Static	1.630e-01	8.281e-02	0.049
	Time-Dependent	-6.084e-03	6.956e-03	0.381
**Prone position, yes**	Static	-4.407e-02	1.616e+00	0.978
	Time-Dependent	-1.058e-01	1.722e-01	0.539
**Sex, Male**	Static	6.527e+00	6.527e+00	0.298
**Systolic arterial pressure, mmHg**	Static	-5.435e-02	4.555e-02	0.233
	Time-Dependent	2.599e-03	4.569e-03	0.570
**Time, days**	Static	2.650e+00	1.058e+00	0.012
**Tidal volume, ml/kg**	Static	1.552e+00	5.771e-01	0.007
	Time-Dependent	-1.66e-01	5.334e-02	0.002

Abbreviations: APACHE II – Acute Physiology and Chronic Health disease Classification System; ECMO – Extracorporeal membrane oxygenation; FiO_2 – Fraction of inspired oxygen; paO_2 – partial pressure of arterial oxygen; PEEP – Positive end-expiratory pressure; WCE – Weighted cumulative exposure.

e-Figure 6. Duration- and dose-effect relationship between ketamine (propofol) and alkaline phosphatase levels

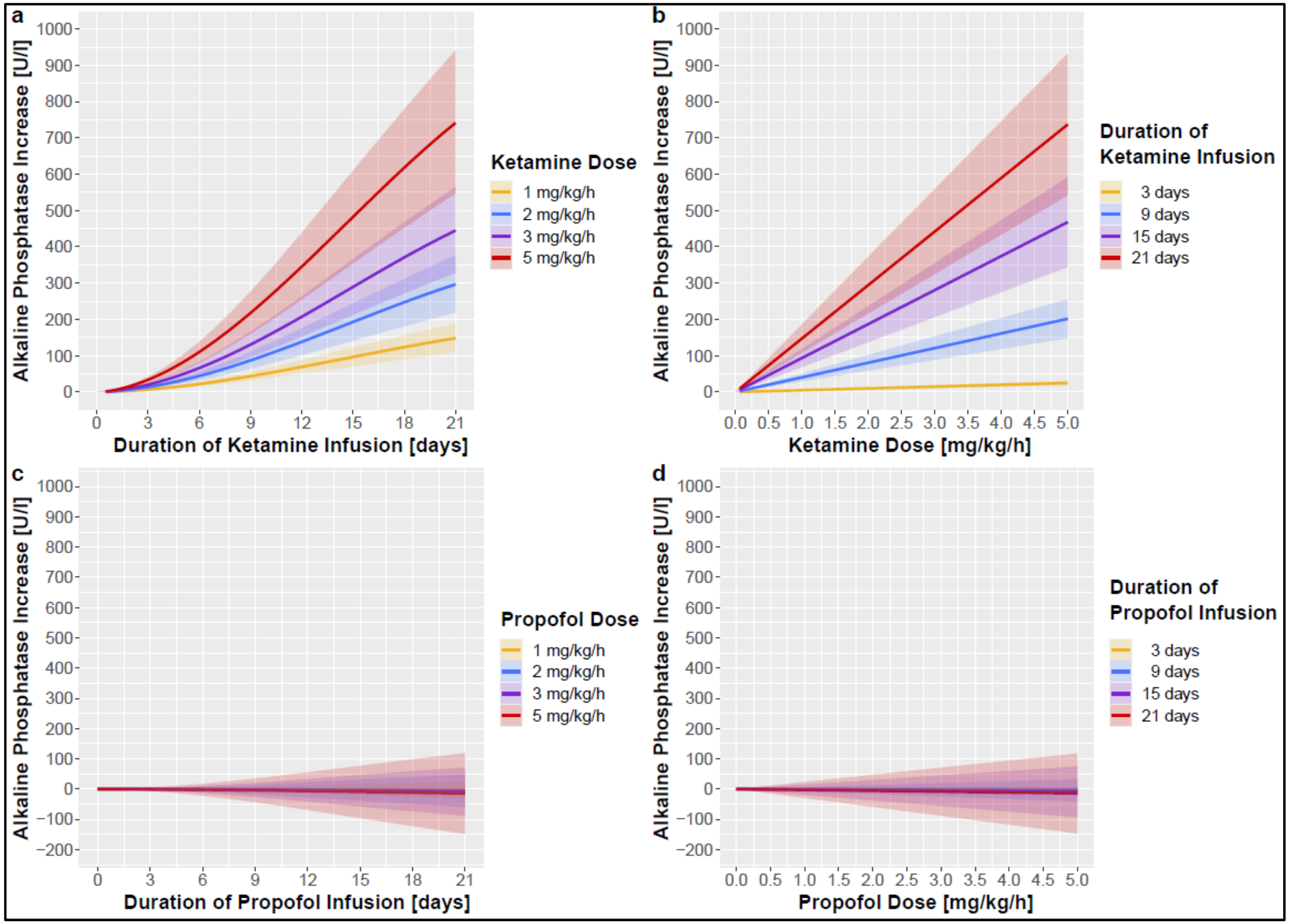

Time-varying, weighted cumulative exposure mixed-effects model assessing the multivariable adjusted duration of infusion-effect **(a)** and dose effect **(b)** relationship of ketamine (propofol **(c, d)**) on rising alkaline phosphatase levels. Model estimates are depicted as solid lines and 95% confidence intervals as shaded areas.

e-Table 6. Time-varying weighted cumulative exposure mixed-effects model for alkaline phosphatase

	Variance		Standard Deviation	
**Random Effect**				
Intercept	19090.6		138.17	
Slope	257.1		16.04	
	**Interaction**	**Estimate**	**Standard Error**	**p**
**Fixed Effects**				
Intercept	Static	3.533e+02	7.653e+01	<0.0001
**Ketamine, mg/kg/d**	WCE	2.302e-02	3.106e-03	<0.0001
**Propofol, mg/kg/d**	WCE	-4.516e-04	2.103e-03	0.753
**Sufentanil, µg/kg/d**	WCE	-1.122e+00	1.178e+01	0.818
**Age, years**	Static	-1.731e+00	8.299e-01	0.038
**APACHE II Score**	Static	1.228e+00	1.710e+00	0.473
**Central venous pressure, mmHg**	Static	1.259e-01	1.429e-01	0.378
	Time-Dependent	-2.459e-02	1.290e-02	0.057
**C-reactive protein, mg/l**	Static	-2.235e-02	4.641e-02	0.630
	Time-Dependent	1.393e-03	4.946e-03	0.778
**Creatinine, µmol/l**	Static	4.920e-02	7.376e-02	0.505
	Time-Dependent	-2.306e-02	8.925e-03	0.009
**ECMO, yes**	Static	2.456e+01	1.890e+01	0.193
	Time-Dependent	-4.083e+00	1.670e+00	0.015
**FiO_2, %**	Static	3.865e-01	2.470e-01	0.118
	Time-Dependent	2.204e-02	2.760e-02	0.425
**Haemoglobin, g/l**	Static	-6.752e-01	2.964e-01	0.023
	Time-Dependent	-1.768e-01	3.591e-02	<0.0001
**Norepinephrine dose, mg/kg/d**	Static	1.245e+02	3.229e+01	<0.001
	Time-Dependent	4.744e+00	3.352e+00	0.157
**paO_2, kPa**	Static	-2.748e+00	1.997e+00	0.169
	Time-Dependent	1.375e-01	1.650e-01	0.405
**PEEP, cmH_2O**	Static	-4.462e+00	1.644e+00	0.007
	Time-Dependent	1.531e-01	1.636e-01	0.349
**Procalcitonin, µg/l**	Static	3.910e-01	4.631e-01	0.399
	Time-Dependent	-3.221e-02	4.025e-02	0.424
**Prone position, yes**	Static	3.236e-01	9.377e+00	0.972
	Time-Dependent	4.914e-01	1.012e+00	0.627
**Sex, Male**	Static	-4.948e+00	2.151e+01	0.818
**Systolic arterial pressure, mmHg**	Static	-3.856e-01	2.652e-01	0.146
	Time-Dependent	6.121e-02	2.688e-02	0.022
**Time, days**	Static	-1.790e+01	6.306e+00	0.005
**Tidal volume, ml/kg**	Static	3.636e+00	3.324e+00	0.274
	Time-Dependent	-6.869e-01	3.130e-01	0.028

Abbreviations: APACHE II – Acute Physiology and Chronic Health disease Classification System; ECMO – Extracorporeal membrane oxygenation; FiO_2 – Fraction of inspired oxygen; paO_2 – partial pressure of arterial oxygen; PEEP – Positive end-expiratory pressure; WCE – Weighted cumulative exposure.

e-Figure 7. Duration- and dose-effect relationship between ketamine (propofol) and total bilirubin levels in mechanically ventilated patients without ECMO

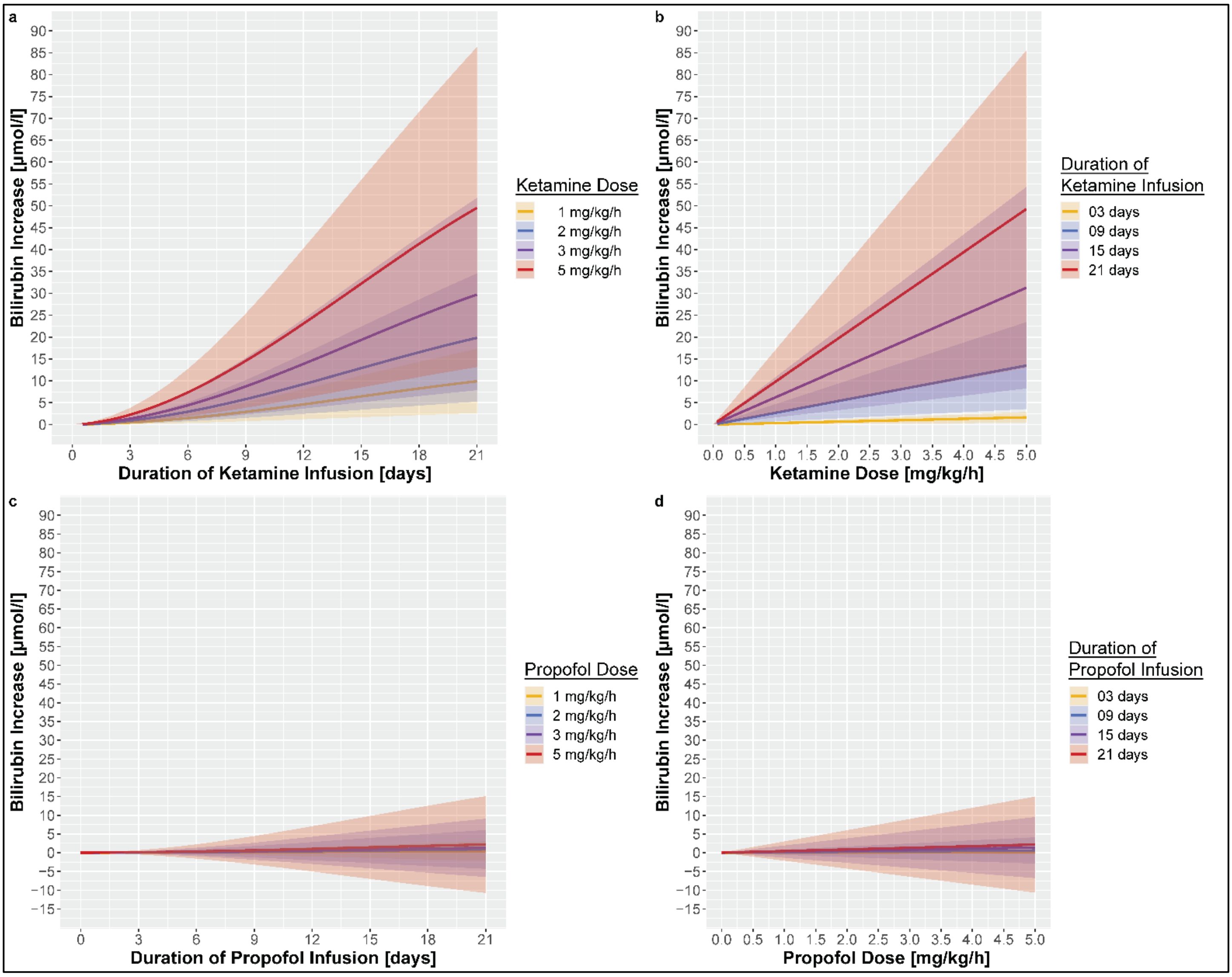

Time-varying, weighted cumulative exposure mixed-effects model assessing the multivariable adjusted duration of infusion-effect **(a)** and dose effect **(b)** relationship of ketamine (propofol **(c, d)**) on rising bilirubin levels. Model estimates are depicted as solid lines and 95% confidence intervals as shaded areas. Sub-cohort of patients not having received extracorporeal membrane oxygenation.

	Estimate	Standard Error	p
**Fixed Effects**			
**Ketamine WCE, mg/kg/d**	1.538e-03	5.773e-04	0.008
**Propofol WCE, mg/kg/d**	6.802e-05	2.036e-04	0.738

e-Figure 8. Duration- and dose-effect relationship between ketamine (propofol) and alkaline phosphatase levels in mechanically ventilated patients without ECMO

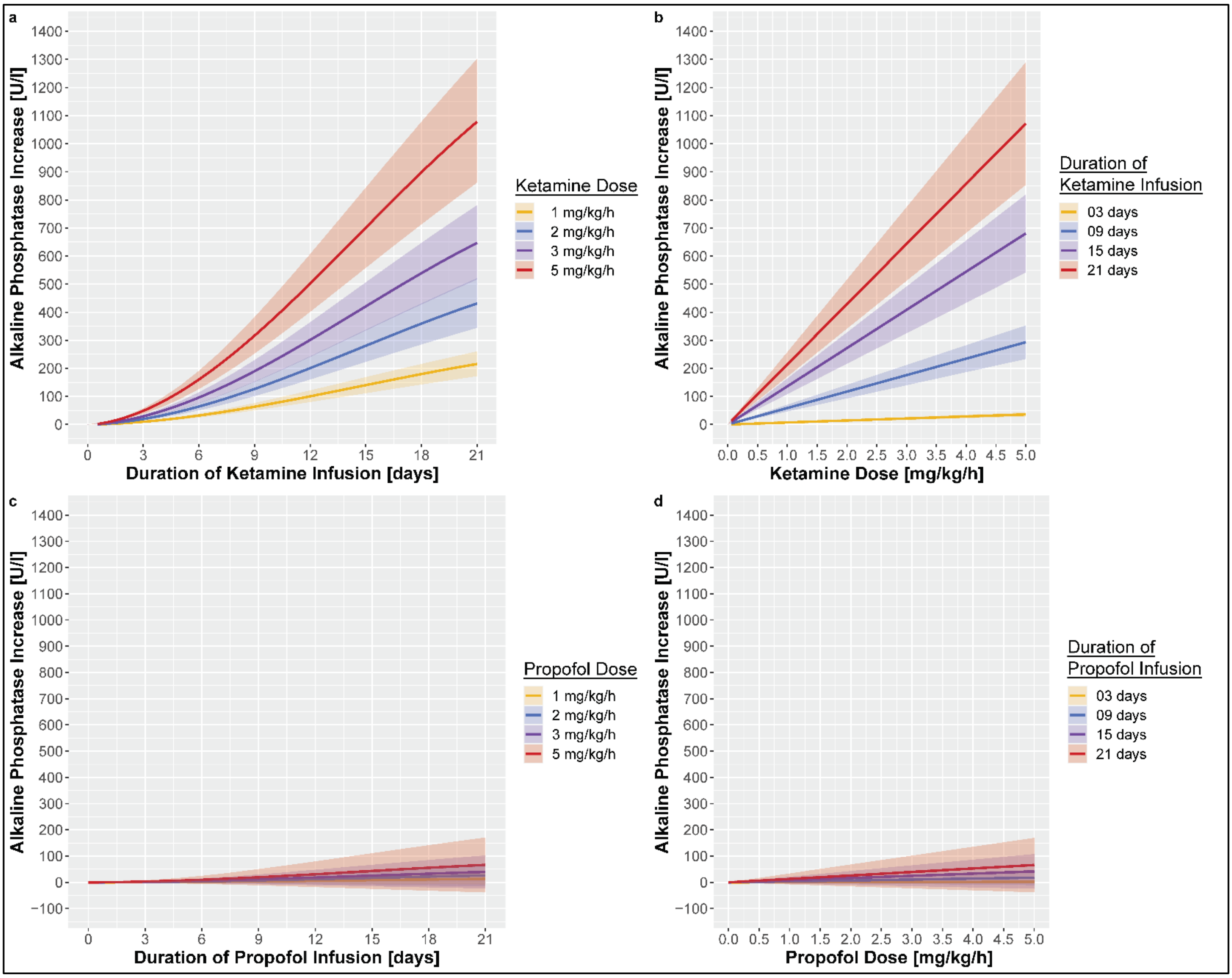

Time-varying, weighted cumulative exposure mixed-effects model assessing the multivariable adjusted duration of infusion-effect **(a)** and dose effect **(b)** relationship of ketamine (propofol **(c, d)**) on rising alkaline phosphatase levels. Model estimates are depicted as solid lines and 95% confidence intervals as shaded areas. Sub-cohort of patients not having received extracorporeal membrane oxygenation.

	Estimate	Standard Error	p
**Fixed Effects**			
**Ketamine WCE, mg/kg/d**	3.347e-02	3.475e-03	<0.0001
**Propofol WCE, mg/kg/d**	2.053e-03	1.648e-03	0.213

e-Table 7. Cut-offs for average daily infusion rate and duration of ketamine infusion for different increases in bilirubin and alkaline phosphatase levels.

	Average daily ketamine infusion rate					
	0.5 mg/kg/h	1.0 mg/kg/h	1.5 mg/kg/h	2.0 mg/kg/h	2.5 mg/kg/h	3.0 mg/kg/h
Increase in Bilirubin [µmol/l]						
**5**	13 days	8 days	7 days	6 days	5 days	5 days
**10**		13 days	10 days	9 days	8 days	7 days
**15**		17 days	13 days	11 days	10 days	9 days
**20**			16 days	13 days	11 days	10 days
**25**			19 days	15 days	13 days	12 days
**30**				17 days	15 days	13 days
**35**				20 days	17 days	15 days
**40**					18 days	16 days
**45**					20 days	18 days
**50**						19 days
**55**						21 days

	Average daily ketamine infusion rate					
	0.5 mg/kg/h	1.0 mg/kg/h	1.5 mg/kg/h	2.0 mg/kg/h	2.5 mg/kg/h	3.0 mg/kg/h
Increase in Alkaline Phosphatase [U/l]						
**50**	15 days	10 days	8 days	7 days	6 days	5 days
**100**		16 days	12 days	10 days	9 days	8 days
**150**		21 days	15 days	13 days	11 days	10 days
**200**			19 days	16 days	13 days	12 days
**250**				18 days	15 days	14 days
**300**				21 days	18 days	16 days
**350**					20 days	17 days
**400**						19 days
**450**						21 days

The combinations of ketamine infusion rate and duration to reach a specific increase in bilirubin or alkaline phosphatase, independently associated with ketamine, were predicted employing the estimates of the time-varying, weighted cumulative exposure mixed-effects models.

e-Table 8. Incidence of cholestatic liver injury, organ support and outcomes

	Overall	No Ketamine Infusion	Ketamine Infusion	p
n	243	73	170	
**Cholestatic Liver Injury**				
None	129 (53)	59 (81)	70 (41)	<0.001
Mild*	80 (33)	13 (18)	67 (39)	
Severe†	34 (14)	1 (1)	33 (19)	
Time from intubation to cholestatic liver injury, days	5 [0, 11]	1.0 [0, 4]	6.0 [1, 12]	0.039
**Organ support and drug therapies**				
Continuous renal replacement therapy	52 (21)	9 (12)	43 (25)	0.037
Extracorporeal membrane oxygenation	42 (17)	1 (1)	41 (24)	<0.001
Corticosteroids	243 (100)	73 (100)	170 (100)	1
Remdesivir	94 (39)	24 (33)	70 (41)	0.283
Tocilizumab	14 (6)	2 (3)	12 (7)	0.306
**Outcomes**				
Length of mechanical ventilation, days	11 [5, 22]	5 [3, 10]	15 [8, 25]	<0.001
Length of intensive care unit stay, days	13 [7, 27]	7 [3, 12]	18 [10, 32]	<0.001
Length of hospital stay, days	22 [14, 40]	16 [10, 25]	26 [17, 44]	<0.001
Intensive care unit survival	180 (74)	60 (82)	120 (71)	0.083
Hospital survival	173 (71)	57 (78)	116 (68)	0.162

Quantitative data are expressed as median [interquartile range] or counts (percentages) as appropriate.

*Mild: Alkaline phosphatase ≥1.5 times the upper limit of normality and Gamma-glutamyltransferase ≥3 times the upper limit of normality.

†Severe: Alkaline phosphatase ≥1.5 times the upper limit of normality, Gamma-glutamyltransferase ≥3 times the upper limit of normality and total bilirubin ≥2 times the upper limit of normality.

e-Figure 9. Cumulative incidence functions for cholestatic liver injury and death

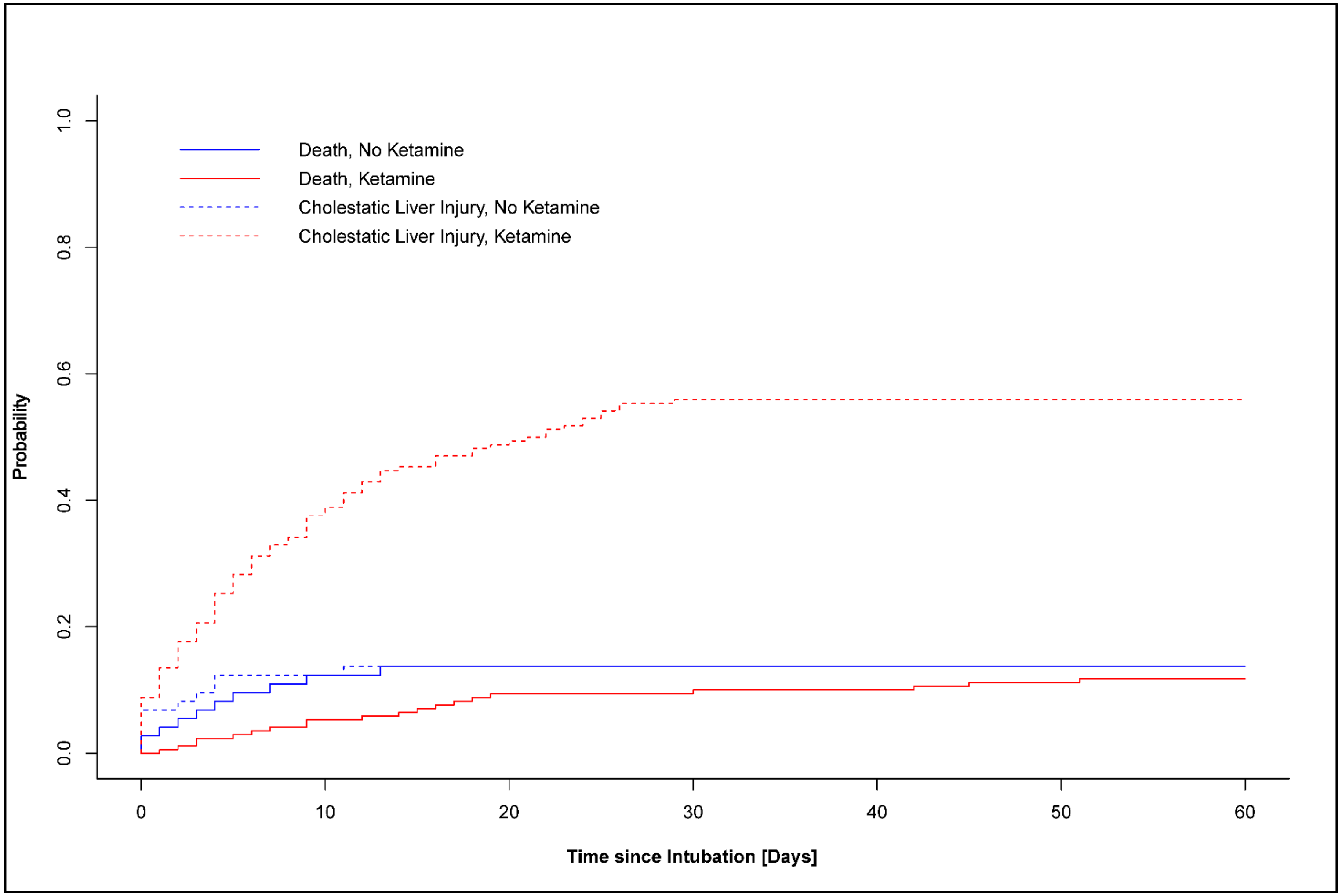

e-Table 9. Multivariable Fine and Gray competing risk proportional hazards model for the incidence of cholestatic liver injury accounting for death

	$Estimate_{exp}$	95% Confidence Interval	p
**Ketamine Infusion, yes**	3.2	1.31 – 7.77	0.01
**Age, years**	1.00	0.98 – 1.02	0.820
**APACHE II Score**	1.00	0.95 – 1.05	0.970
**Bilirubin baseline, µmol/l**	1.01	1.01 – 1.02	<0.0001
**Central venous pressure, mmHg**	1.00	0.997 – 1.01	0.630
**C-reactive protein, mg/l**	1.00	1.00 – 1.00	0.043
**Creatinine, µmol/l**	1.00	0.99 – 1.00	0.350
**ECMO, yes**	1.72	0.71 – 4.14	0.230
**Haemoglobin, g/l**	0.99	0.976 – 1.00	0.052
**Norepinephrine dose, mg/kg/d**	0.25	0.038 – 1.58	0.140
**paO_2/ FiO_2, mmHg**	1.00	0.998 – 1.01	0.294
**PEEP, cmH_2O**	0.98	0.89 – 1.07	0.610
**Procalcitonin, µg/l**	1.01	0.998 – 1.02	0.140
**Prone position, yes**	0.97	0.58 – 1.62	0.900
**Sex, Male**	0.79	0.46 – 1.35	0.390
**SOFA Score**	1.08	0.94 – 1.24	0.280
**Static compliance, ml/cmH_2O**	1.00	0.99 – 1.02	0.770
**Systolic arterial pressure, mmHg**	1.00	0.98 – 1.01	0.730

Abbreviations: APACHE II – Acute Physiology and Chronic Health disease Classification System; ECMO – Extracorporeal membrane oxygenation; FiO_2 – Fraction of inspired oxygen; paO_2 – partial pressure of arterial oxygen; PEEP – Positive end-expiratory pressure; SOFA – Sequential Organ Failure Assessment.

e-Figure 10. Cumulative incidence functions for severe cholestatic liver injury and death

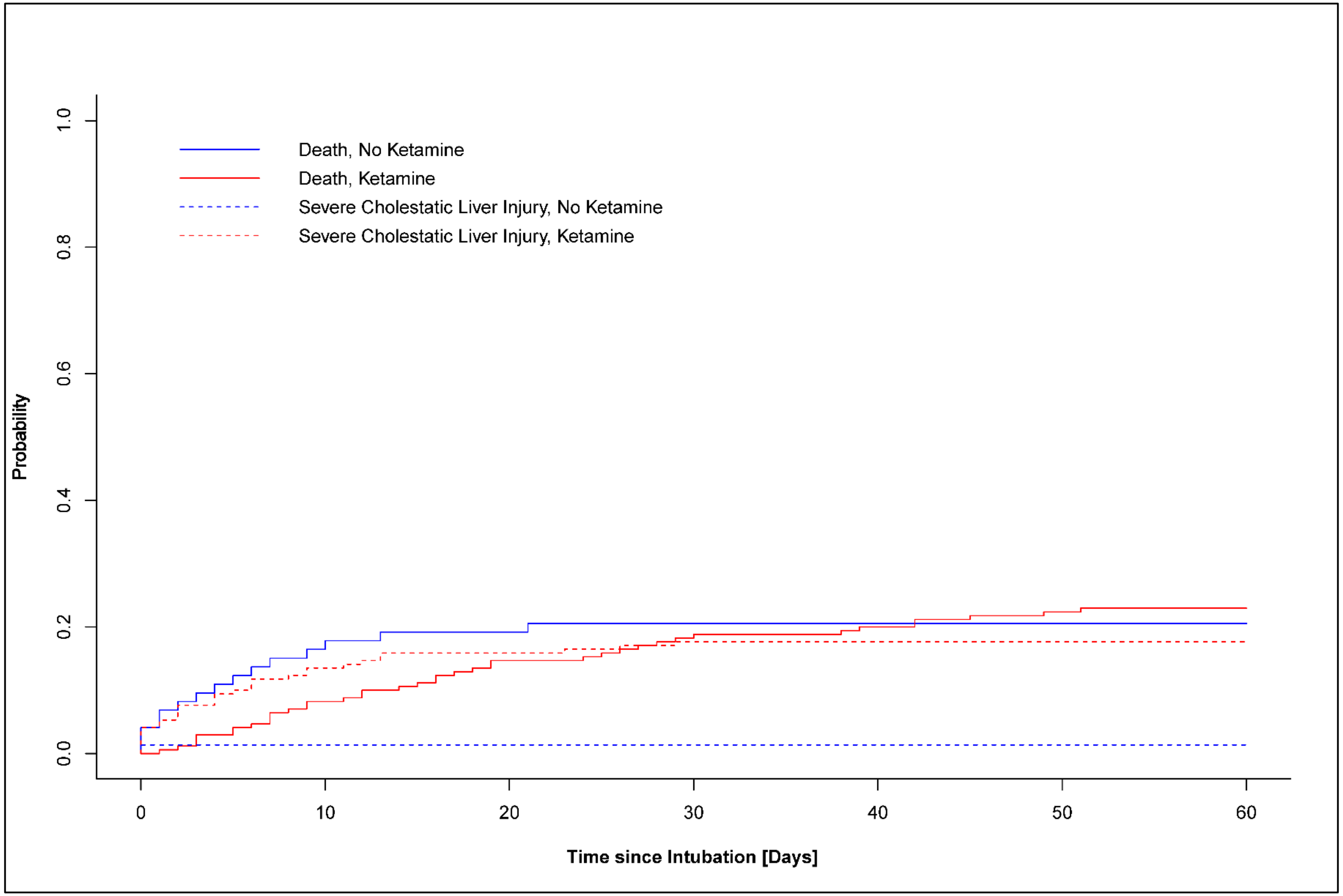

e-Figure 11. Kaplan-Meier and Cox proportional hazards model for hospital mortality

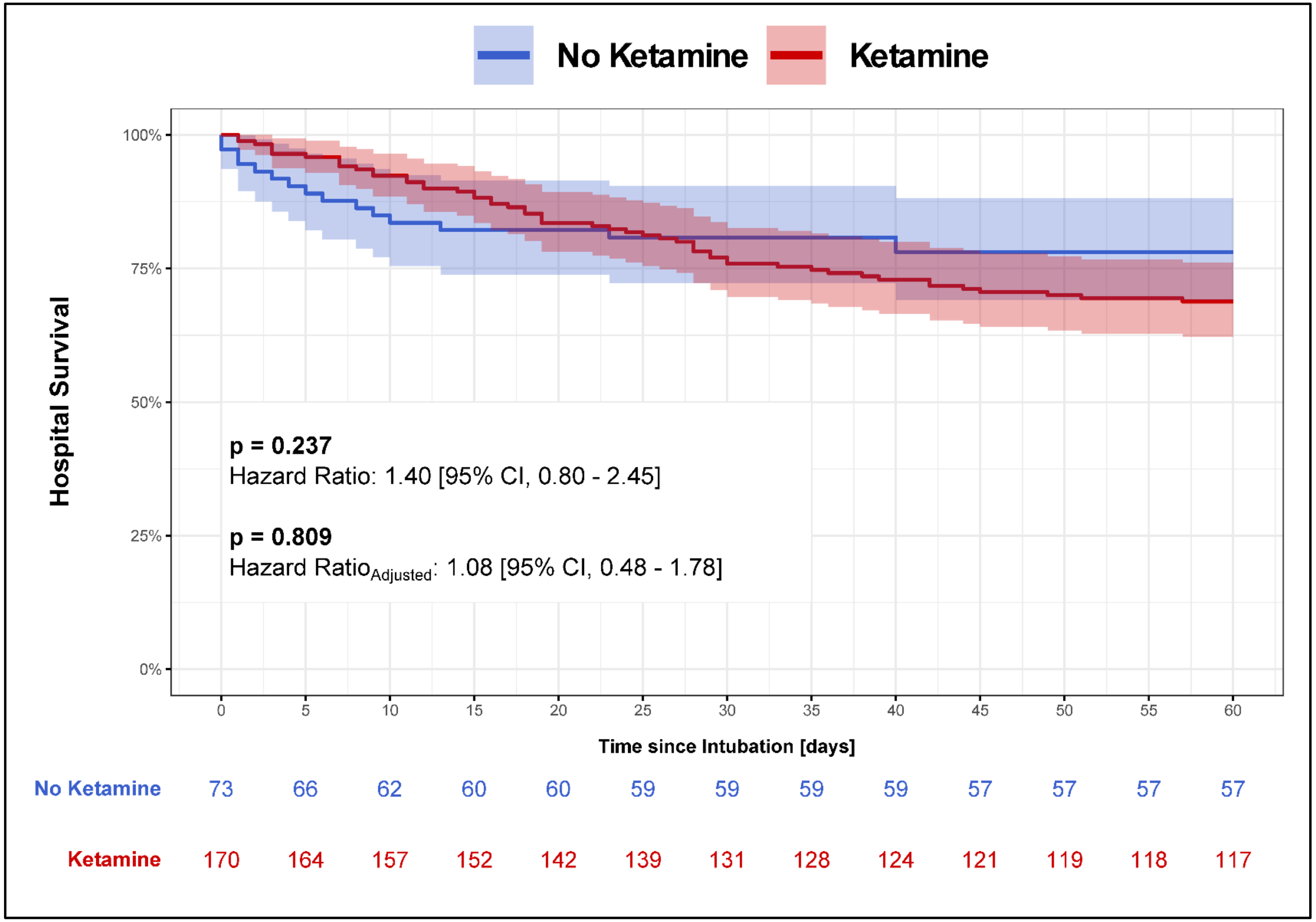

Kaplan–Meier curves for 60-day hospital survival by ketamine infusion. Shaded areas represent the crude 95% confidence intervals. The computed hazard ratio assesses the risk of ketamine infused patients against those not having received it. The 95% confidence interval is given in parentheses. Crude and multivariable adjusted hazard ratios are depicted. The underlying table presents the patients at risk per time point.

e-Table 10. Multivariable Cox proportional hazards model for hospital survival

	$Estimate_{exp}$	95% Confidence Interval	p
**Ketamine Infusion, yes**	0.69	0.33 – 1.46	0.332
**Age, years**	1.08	1.04 – 1.12	<0.0001
**APACHE II Score**	0.98	0.91 – 1.06	0.657
**Bilirubin baseline, µmol/l**	1.01	0.998 – 1.02	0.140
**Central venous pressure, mmHg**	0.97	0.92 – 1.03	0.288
**C-reactive protein, mg/l**	1.00	0.995 – 1.00	0.213
**Creatinine, µmol/l**	1.00	0.998 – 1.01	0.337
**ECMO, yes**	2.47	1.16 – 5.30	0.02
**Haemoglobin, g/l**	1.00	0.99 – 1.02	0.551
**Norepinephrine dose, mg/kg/d**	7.86	1.76 – 35.15	0.007
**paO_2/ FiO_2, mmHg**	1.00	0.99 – 1.00	0.541
**PEEP, cmH_2O**	0.97	0.88 – 1.08	0.611
**Procalcitonin, µg/l**	1.00	0.99 – 1.02	0.758
**Sex, Male**	0.92	0.46 – 1.87	0.823
**SOFA Score**	1.19	1.02 – 1.40	0.029
**Static compliance, ml/cmH_2O**	1.00	0.98 – 1.02	0.952
**Systolic arterial pressure, mmHg**	1.00	0.98 – 1.02	0.782

Abbreviations: APACHE II – Acute Physiology and Chronic Health disease Classification System; ECMO – Extracorporeal membrane oxygenation; FiO_2 – Fraction of inspired oxygen; paO_2 – partial pressure of arterial oxygen; PEEP – Positive end-expiratory pressure; SOFA – Sequential Organ Failure Assessment.

e-Appendix 4. Bradford Hill Criteria for causal inference in epidemiological association studies

The nine Bradford Hill criteria were published by Sir Austin Bradford Hill in 1965 as a means to aid in determining if associations observed in epidemiological studies underlie a causal relationship [7]. The Bradford Hill criteria have since then become a mayor tool to assert causal inference in epidemiology [8]. They represent a flexible guideline, and not all considerations can, or have to be met in order to determine causality [8].

Bradford Hill Criterium	Evidence provided by this study	Other evidence
**1. Strength of association**	• Strong association between cumulative doses of ketamine and maximal bilirubin doses.<br>• Very strong association between crude and multivariable adjusted hazard of cholestatic liver injury, especially ints severe expression.	• Strong association between ketamine abuse and incidence of cholangiopathy [9].
**2. Consistency**	• Consistency of association across markers of cholestatic liver injury (bilirubin, alkaline phosphatase).	• Consistency across cohorts: COVID-19 ARDS [10-12], burns ARDS [13], ketamine abusers [9], pain therapy over long periods [14].
**3. Specificity**	• Specificity of relationship to cholestatic liver injury and specifically to ketamine (no association with long-term propofol infusion).	• Systematical reporting of cholangiopathies isolated from other forms of hepatopathies [9].<br>• No association with other sedative and analgetic agents [12].
**4. Temporality**	• Duration-effect and cumulative dose-effect relationship reflected in two markers of cholestatic live injury.<br>• Much larger hazard for cholestatic liver injury post ketamine infusion.	• Association only in longterm abuse or infusion scenarios [9, 11-14], not in shortterm scenarios [15].<br>• Reversibility of cholangiopathy after halting of ketamine abuse [9].
**5. Biological gradient**	• Clear dose-effect relationship	• Reversibility of cholangiopathy after halting of ketamine abuse [9].
**6. Plausibility**	• Two distinct markers of cholestatic liver injury presenting the same dose-effect relationship	• Multiple biological pathways postulated, however still no clear biological evidence [16-18].
**7. Coherence**	• Coherent effect across biomarkers and without overlap over different classes of anaesthetic agents.	• Imaging asserted changes in the bile ducts of ketamine abusers [9].<br>• Increased incidence of cholangiopathies in patients suffering from ARDS since ketamine has been used as a long-term analgosedative agent [11-13].
**8. Experiment**	• Causal integration framework, based on a prespecified structural causal model, and a statistical model showing robust evidence for the cause-effect relationship in this framework.	• Increased incidence of cholangiopathies in ketamine abusers [9].
**9. Analogy**		• Other drugs have been associated to cholangiopathies, such as chemotherapeutics or antibiotics. However, none share pathways with ketamine.

References

1. Jick H, Rodriguez LAG, Perez-Gutthann S: **Principles of epidemiological research on adverse and beneficial drug effects**. *The Lancet* 1998, **352**(9142):1767-1770.
2. Abrahamowicz M, Bartlett G, Tamblyn R, du Berger R: **Modeling cumulative dose and exposure duration provided insights regarding the associations between benzodiazepines and injuries**. *Journal of Clinical Epidemiology* 2006, **59**(4):393-403.
3. Stranges S, Bonner MR, Fucci F, Cummings KM, Freudenheim JL, Dorn JM, Muti P, Giovino GA, Hyland A, Trevisan M: **Lifetime Cumulative Exposure to Secondhand Smoke and Risk of Myocardial Infarction in Never Smokers: Results From the Western New York Health Study, 1995-2001**. *Archives of Internal Medicine* 2006, **166**(18):1961-1967.
4. Danieli C, Sheppard T, Costello R, Dixon WG, Abrahamowicz M: **Modeling of cumulative effects of time-varying drug exposures on within-subject changes in a continuous outcome**. *Statistical Methods in Medical Research* 2020, **29**(9):2554-2568.
5. Sylvestre M-P, Abrahamowicz M: **Flexible modeling of the cumulative effects of time-dependent exposures on the hazard**. *Statistics in Medicine* 2009, **28**(27):3437-3453.
6. Vacek PM: **ASSESSING THE EFFECT OF INTENSITY WHEN EXPOSURE VARIES OVER TIME**. *Statistics in Medicine* 1997, **16**(5):505-513.
7. Hill AB: **The envirnonment and disease: Association or Causation?** *Proc R Soc Med* 1965, **58**(5):295-300.
8. Fedak KM, Bernal A, Capshaw ZA, Gross S: **Applying the Bradford Hill criteria in the 21st century: how data integration has changed causal inference in molecular epidemiology**. *Emerging Themes in Epidemiology* 2015, **12**(1):14.
9. Seto W-K, Mak S-K, Chiu K, Vardhanabhuti V, Wong H-F, Leong H-T, Lee PSF, Ho YC, Lee C-K, Cheung K-S *et al*: **Magnetic resonance cholangiogram patterns and clinical profiles of ketamine-related cholangiopathy in drug users**. *Journal of Hepatology* 2018, **69**(1):121-128.

10. Bütikofer S, Lenggenhager D, Wendel Garcia PD, Maggio EM, Haberecker M, Reiner CS, Brüllmann G, Buehler PK, Gubler C, Müllhaupt B *et al*: **Secondary sclerosing cholangitis as cause of persistent jaundice in patients with severe COVID-19**. *Liver International* 2021, **n/a**(n/a).

11. Mallet V, Bock K, Mandengue PD, Dufour N, Voigtlaender T, Ricard J-D, Isnard P, Frochot V, Letavernier E, Moga L *et al*: **Intravenous ketamine and progressive cholangiopathy in COVID-19 patients**. *Journal of Hepatology* 2021, **74**(5):1243-1244.

12. Mallet V, Mallet V, Bock K, Dellagi M, Tano M, Siorat V, Beeker N, Paubel P, Mandengue PD, Dufour N *et al*: **Reply to: Progressive cholangiopathy in COVID-19 patients: Other possible diagnoses than ketamine-induced cholangiopathy should be considered&#x201d**. *Journal of Hepatology* 2021, **75**(4):990-992.

13. de Tymowski C, Dépret F, Dudoignon E, Legrand M, Mallet V, Mallet V, Bock K, Mandengue PD, Dufour N, Ricard J-D *et al*: **Ketamine-induced cholangiopathy in ARDS patients**. *Intensive Care Medicine* 2021.

14. Noppers IM, Niesters M, Aarts LPHJ, Bauer MCR, Drewes AM, Dahan A, Sarton EY: **Drug-induced liver injury following a repeated course of ketamine treatment for chronic pain in CRPS type 1 patients: A report of 3 cases**. *PAIN* 2011, **152**(9).

15. Garber PM, Droege CA, Carter KE, Harger NJ, Mueller EW: **Continuous Infusion Ketamine for Adjunctive Analgosedation in Mechanically Ventilated, Critically Ill Patients**. *Pharmacotherapy: The Journal of Human Pharmacology and Drug Therapy* 2019, **39**(3):288-296.

16. Bokor G, Anderson PD: **Ketamine: An Update on Its Abuse**. *Journal of Pharmacy Practice* 2014, **27**(6):582-586.

17. Lo RS, Krishnamoorthy R, Freeman JG, Austin AS: **Cholestasis and biliary dilatation associated with chronic ketamine abuse: a case series**. *Singapore Med J* 2011, **52**(3):e52-55.

18. Thune A, Jivegård L, Pollard H, Moreau J, Schwartz JC, Svanvik J: **Location of enkephalinase and functional effects of [Leu5] enkephalin and inhibition of enkephalinase in the feline main pancreatic and bile duct sphincters**. *Clinical Science* 1992, **82**(2):169-173.
